# Identification and validation of biomarkers related to centrosome replication in ulcerative colitis based on bulk transcriptome, single-cell RNA sequencing and experiments

**DOI:** 10.3389/fimmu.2025.1627926

**Published:** 2025-12-12

**Authors:** Zhenhuan Yang, Xingxing Wu, Lei Luo, Xiuxia Wu, Yuliang Wang, Tingting Huang, Zhongqin Dang, Shanwen Nie

**Affiliations:** Department of Gastroenterology, Henan Provincial Hospital of Traditional Chinese Medicine (The Second Affiliated Hospital of Henan University of Traditional Chinese Medicine), Zhengzhou, Henan, China

**Keywords:** ulcerative colitis, centrosome amplification, biomarkers, machine learning, single-cell RNA sequencing

## Abstract

**Background:**

Ulcerative colitis (UC) is a complex chronic inflammatory disease. Centrosome amplification (CA) has been implicated in UC pathogenesis, but its mechanistic role remains unclear. This study aimed to investigate the relevance of centrosome amplification-related genes (CARGs) in UC progression.

**Methods:**

UC and control samples, along with CARGs, were obtained from public databases. Differential expression analysis identified differentially expressed genes (DEGs) between UC and controls. Candidate genes were selected by intersecting DEGs with CARGs. Biomarker identification employed 11 machine learning algorithms, receiver operating characteristic (ROC) analysis, and expression validation. Functional insights were gained through gene set enrichment analysis (GSEA), immune infiltration profiling, and clustering analysis. Cellular expression patterns of biomarkers were also examined. Finally, biomarker expression in colonic mucosal tissue was validated by RT-qPCR, Western blot, and Immunohistochemistry.

**Results:**

Six biomarkers—TEX11, SLC16A1, OVOL1, EDNRA, HEPACAM2, and SPIRE2—were identified. Enriched pathways associated with these genes included cell adhesion molecules (CAMs) and oxidative phosphorylation. Immune infiltration analysis revealed significant interactions between biomarkers and differential immune cells (DICs), such as neutrophils, in UC. Consensus clustering stratified UC samples into two clusters, with DICs including M0 macrophages showing significant correlations with biomarkers. Single-cell expression analysis highlighted undifferentiated and enteroendocrine cells as potentially key cell types in UC. Validation through animal and clinical experiments demonstrated downregulation of SLC16A1, OVOL1, TEX11, and HEPACAM2, alongside upregulation of EDNRA in colonic mucosa of UC compared to controls.

**Conclusion:**

Six CARGs—TEX11, SLC16A1, OVOL1, EDNRA, HEPACAM2, and SPIRE2—were identified as potential biomarkers with significant implications in UC pathogenesis.

## 1 Introduction

Ulcerative colitis (UC) is a subtype of inflammatory bowel disease (IBD) characterized by persistent and recurring inflammation of the colonic mucosa (1). Common clinical manifestations typically include ongoing or recurring diarrhea, mucus and bloody stools, along with systemic symptoms of varying severity (2). As colonoscopy screening becomes more widespread and lifestyle and dietary habits evolve, the incidence and prevalence of UC have been rising annually on a global scale (3). Despite the growing number of treatment options, managing UC remains a significant challenge (4). The pathogenesis of UC is still unclear (5, 6). Conducting comprehensive research on the molecular mechanisms of UC and identifying biomarkers for disease progression may provide new insights for early diagnosis and treatment.

Abnormal changes in the size, shape, number, and location of the centrosome are collectively referred to as centrosome amplification (CA) (7). CA results from several mechanisms, including cell division failure, improper regulation of centrosome replication proteins, and fragmentation of pericentriolar material (8). As anticipated, CA has been detected in various human cancers, often linked to abnormal chromosomal configurations, genomic instability, disease progression, and poor patient outcomes (9–11). Several human diseases, including those caused by oncogenic viruses, type 2 diabetes, and inflammatory diseases, have been associated with CA (12). However, the molecular mechanisms of CA in UC remain largely unexplored.

Single-cell RNA sequencing (scRNA-seq) has driven substantial progress in mammalian research, enhancing our understanding of transcriptional diversity across various cell types and states (13, 14). This technology enables an unbiased exploration of the molecular underpinnings and consequences of cellular heterogeneity. Research indicates that the ethyl acetate extract of Sanguisorba officinalis mitigates UC by inhibiting the PI3K-AKT/NF-κB/STAT3 pathway, as demonstrated through scRNA-seq analysis (15). The identification of lncRNAs linked to tumor-infiltrating immune cells through machine learning contributes to better clinical outcomes and boosts the efficacy of immunotherapy in individuals with low-grade glioma (16). Both single-cell and bulk RNA sequencing have identified fibroblast signatures and CD8+ T-cell–fibroblast subtypes, which act as promising biomarkers for forecasting immunotherapy response in bladder cancer (17).

In this study, a novel computational framework integrated with 11 machine learning algorithms (113 combinations) was employed to identify biomarkers linked to centrosome replication in UC. UC and control samples, as well as CA-related genes (CARGs), were retrieved from public databases. DEGs between UC and controls were identified through differential expression analysis. Candidate genes were determined through the intersection of DEGs and CARGs. Subsequently, 11 machine learning algorithms (113 combinations), ROC analysis, and expression verification were applied to filter relevant biomarkers. The potential molecular mechanisms associated with centrosome replication in UC were further explored through bioinformatics analyses, including GSEA, immune infiltration analysis, clustering analysis, transcription factor and microRNA (miRNA) predictions, and scRNA-seq analysis, offering valuable insights for the management of UC.

## 2 Methods

### 2.1 Data collection

Transcriptome data for UC (GSE87473, GSE75214, GSE92415, GSE87466, and GSE116222) were retrieved from the Gene Expression Omnibus (GEO) database (https://www.ncbi.nlm.nih.gov/geo/). The GSE87473 dataset (GPL13158) comprised 106 UC and 21 control colonic mucosal tissue samples. The GSE75214 dataset (GPL6244) included 74 active UC and 11 control colonic mucosal tissue samples. The GSE92415 dataset (GPL13158) contained 53 colon mucosal tissue samples from patients with UC treated with placebo. The GSE87466 dataset (GPL13158) comprised 87 UC and 21 control colonic mucosal tissue samples, while the GSE116222 dataset (GPL24676) involved 3 inflamed UC and 3 control intestinal epithelial cell samples. It should be noted that GSE87473 was used as the primary training set; GSE87473 was used as the training dataset for the machine learning model, and GSE75214 was used as the tuning dataset for machine learning; GSE87466 were used for expression level validation and ROC analysis; GSE92415 was used for analyzing the Mayo score of UC; and GSE116222 was used for conducting single-cell analysis. CARGs were obtained by searching for “centrosome amplification” in the Gene Ontology (GO, http://geneontology.org/) and Kyoto Encyclopedia of Genes and Genomes (KEGG, https://www.kegg.jp/) databases (**Supplementary Table S1**).

### 2.2 Identification of DEGs and candidate genes

First, the limma package (v 3.58.1) (18) was used to perform log_2_ CPM conversion and weight calculation. For all samples in GSE87473, the “limma” package (v 3.58.1) was used to identify DEGs between UC and control samples (UC vs. control) with |log_2_FoldChange (FC)| > 1 and *P* < 0.05. Based on Based on the log_2_FC value, a volcano plot created using the “ggplot2” package (v 3.5.1) (19) visualized the DEGs, with the top 10 upregulated and downregulated genes labeled. Additionally, a heatmap generated using the “ComplexHeatmap” package (v 2.18.0) (20) displayed the top 10 upregulated and downregulated genes between the two groups. Meanwhile, a sensitivity analysis was conducted to evaluate robustness of the selected threshold. Candidate genes were selected by intersecting DEGs and CARGs using the “ggvenn” package (v 0.1.10) (21). The “OmicCircos” package (v 1.2.2) (22) was then applied to visualize the distribution of biomarkers across chromosomes.

### 2.3 Enrichment analysis

GO and KEGG enrichment analyses were conducted to examine the biological functions and pathways associated with candidate genes, utilizing the “clusterProfiler” package (v 4.10.1) (23) and the “GOplot” package (v 1.0.2) (24). The top five most significantly enriched GO terms and all KEGG pathways were visualized.

### 2.4 Construction of 113 machine learning models and identification of biomarkers

The GSE87473 and GSE75214 datasets were analyzed using a leave-one-out cross-validation (LOOCV) framework, integrating 11 machine learning algorithms for a total of 113 combinations. Candidate genes were used as input, and the algorithms included: Random Survival Forest (RSF) implemented via the caret package (v6.0.94) (25), SVM and NaiveBayes from the e1071 package (v1.7.14) (26), Stepglm and LDA from the MASS package, plsRglm (v1.5.1) (27), LASSO from the glmnet package (v4.1.8) (28), Generalized Boosted Regression Models (GBRM) from the gbm package (v2.1.9) (29), xBoost from the xgboost package (v2.0.3.1) (30), and glmBoost from the mboost package (v2.9.10) (31). A random seed of 99 was set for each combination.

The pROC package (v1.18.5) (32) was used to plot ROC curves and evaluate predictive performance. The optimal model had to meet the following criteria: (1) average AUC ≥ 0.95; (2) standard deviation of AUC in LOOCV < 0.05; and (3) AUC difference between the internal validation set and the training set < 0.05, with AUC not equal to 1. The model with the highest average AUC was selected, and its corresponding genes were identified as candidate biomarkers.

To validate model robustness, the independent dataset GSE87466 was introduced for external validation. The ComplexHeatmap package was used to systematically compare the AUC values of the 113 algorithm combinations across the three datasets, requiring the optimal model to achieve an AUC > 0.7 in the independent validation set. Furthermore, the pROC package was utilized in the three datasets to evaluate the discriminative ability of individual biomarkers, retaining only those with AUCs greater than 0.7 in all three datasets. Wilcoxon tests were employed to analyze the expression levels of these genes, and finally, genes showing significant and consistent expression trends across all three datasets were identified as the final biomarkers.

To gain deeper insights into the contribution patterns of the biomarkers, SHAP analysis was performed using the “shapviz” (v0.9.8) (https://github.com/ModelOriented/shapviz) and “fastshap” (v0.1.1) (https://github.com/bgreenwell/fastshap, https://bgreenwell.github.io/fastshap/) R packages. SHAP values were calculated for samples via 100 Monte Carlo simulations, randomly drawing training set samples to ensure stability. Feature importance was assessed through both the mean absolute SHAP value and the Random Forest built-in Mean Decrease Accuracy metric for dual verification.

### 2.5 GSEA

To investigate the biological functions of biomarkers for UC, GSEA was conducted in this study. The “c2.cp.kegg.v7.4.symbols.gmt” gene set, sourced from the Molecular Signatures Database (https://www.gsea-msigdb.org/gsea/msigdb/), served as the reference gene set. Spearman correlations between each biomarker and other genes were computed using the “psych” package (v 2.4.3) (33) and the genes were ranked based on their correlation coefficients in descending order. GSEA was then performed using the “clusterProfiler” package (v 4.10.1) on all samples in the GSE87473 dataset, and the top 5 pathways were visualized based on *P-*values (*P* < 0.05).

### 2.6 Consistent clustering analysis

To cluster the 106 UC samples into distinct groups in the GSE87473 dataset, based on biomarkers, consistent clustering analysis was executed using the “ConsensusClusterPlus” package, with the maximum number of clusters (maxK) set to 9. The selection criteria for the optimal K-value were as follows: (1) Consensus Matrix: It evaluated the consistency of samples being assigned to the same cluster under the same K-value; the closer the value was to 1, the higher the consistency. (2) CDF Curve (Cumulative Distribution Function Curve): It analyzed the growth trend of CDF under different K-values; when the K-value increased to a certain value, the growth of CDF slowed down, and this value was regarded as the candidate optimal K-value. (3) Tracking Plot: It observed the changes in cluster assignment of samples under different K-values; the smaller the changes were, the more stable the clustering was. The expression of biomarkers across different clusters was analyzed using the Wilcoxon test, and the results were visualized with the “ggplot2” package (v 3.5.1). Additionally, expression heatmaps of biomarkers in different clusters were generated using the “pheatmap” package (v 1.0.12) (34).

### 2.7 Gene set variation analysis

Pathway enrichment scores for the samples in each cluster were calculated using the “GSVA” package (v 1.50.0) (35), with the reference gene set “h.all.v2024.1.Hs.symbols” obtained from the MSigDB database. The “limma” package (v 3.58.1) was applied to identify biological pathways with significant differences between clusters (*P* < 0.05, |t| > 2) in the GSE87473 dataset.

### 2.8 Immune infiltration analysis

The CIBERSORT algorithm (36) was used to analyze the infiltration of 22 immune cell types in all UC and control samples in GSE87473, excluding samples with *P* > 0.05. Differences in immune cell infiltration between clusters were also assessed using the same method, and the results were visualized with the “ggplot2” package (v 3.5.1). The “LM22.txt” reference immune cell expression file was obtained from the CIBERSORT database (https://cibersort.stanford.edu/). Immune cells with zero infiltration in at least 50% of samples were filtered out. DICs between UC and control samples, and between different clusters, were identified using the Wilcoxon test. Correlations between biomarkers and DICs were evaluated using the “psych” package (v 2.4.3) (|R| > 0.3, *P* < 0.05).

### 2.9 Analysis of inflammatory factors and the activity of UC

In this study, cellular inflammatory factors (TNF, IFNG, IL6, IL8, and IL34) were sourced from previous research (37–39). Differential cellular inflammatory factors (DCIFs) in the GSE87473 dataset were identified using the Wilcoxon test. The correlations between biomarkers and DCIFs were calculated using the same method *via* the “psych” package (v 2.4.3) (|R| > 0.3, *P* < 0.05) in the GSE87473 dataset. The Mayo score for UC severity was obtained from the GSE92415 dataset, with higher scores indicating more severe UC. To evaluate the correlation between biomarkers and UC activity, correlation analysis between biomarkers and the Mayo score was performed using the same method (|R| > 0.3, *P* < 0.05) in the GSE92415 dataset.

### 2.10 Prediction of transcription factors and microRNAs

TFs associated with biomarkers were predicted using the miRNet database (https://www.mirnet.ca/). Differential TFs were identified by intersecting DEGs with TFs, and the results were visualized using the “ggvenn” package (v 0.1.10). Network visualization was performed using Cytoscape (v 3.9.1). The cor package was used to analyze the correlation between biomarkers and differentially expressed TFs (|R| > 0.3, *P* < 0.05). Subsequently, miRNAs were predicted using the “multiMiR” package (v 1.24.0) (40) across four databases: miRDB (https://mirdb.org/), miRanda (http://mirtoolsgallery.tech/mirtoolsgallery/node/1055), DIANA-microT (http://mirtoolsgallery.tech/mirtoolsgallery/node/1084), and ElMMo (http://mirtoolsgallery.tech/mirtoolsgallery/node/1098). The miRNAs linked to biomarkers were obtained by overlapping the miRNAs from these four databases, and results were visualized using the “ggvenn” package (v 0.1.10).

### 2.11 Prediction of chemical compounds

To explore chemical compounds associated with biomarkers, the Comparative Toxicogenomics Database (https://ctdbase.org/) was utilized to predict chemical compounds. The top 10 chemical compounds with the highest interaction counts for each biomarker were visualized in the study.

### 2.12 Single-cell RNA-sequencing analysis

Single-cell RNA sequencing (scRNA-seq) analysis of the GSE116222 dataset was performed using Seurat (v5.1.0) (41). Quality control was first conducted using the PercentageFeatureSet function, with results visualized via ggplot2 (v3.5.1). The NormalizeData function was then applied to normalize feature expression measurements for each cell using a scale factor of 10,000 followed by log-transformation. The top 2,000 highly variable genes were identified using the FindVariableFeatures function, and the top 10 genes showing the highest variation were visualized using LabelPoints. After scaling the data with the ScaleData function, principal component analysis (PCA) was performed using RunPCA. The ElbowPlot function was used to determine the number of significant principal components (*P* < 0.05) for cell clustering. Cell clustering was subsequently carried out using the FindNeighbors and FindClusters functions, and results were visualized through RunUMAP (resolution = 0.4). Cell types were annotated based on marker genes reported in the literature (42), and their expression patterns were visualized.

To identify UC-associated cell types, the Kruskal-Wallis test was used to compare differences in cell type proportions across samples, and the Wilcoxon test was applied to analyze differential expression of biomarkers across various cell types between UC and control samples, with results visualized using ggplot2. CellChat (v1.5.0) (43) was employed to infer cell-cell communication networks among all cell types and evaluate potential ligand-receptor interactions. Dimensionality reduction and clustering methods for key cells remained consistent with the aforementioned approaches. Pseudotime analysis was conducted using Monocle2 (v2.22.0) (44) to explore the dynamic expression patterns of biomarkers during cell differentiation.

### 2.13 Construction and evaluation of the UC mouse model induced by DSS

The experiment utilized male C57BL/6 mice of SPF grade, aged 6-8 weeks, with a body weight of 20-22 grams. The animals were obtained from Beijing Sibef Biotechnology Co., Ltd., under the certification number SCXK(Jing)2019-0010. Ethical approval for the research was granted by the Experimental Animal Ethics Committee of Henan Provincial Hospital of Traditional Chinese Medicine (Ref: PZ-HNSZYY-2023-029), and all experimental procedures adhered to the applicable guidelines for animal ethics. The mice were acclimated for one week under controlled conditions. Following acclimatization, the animals were randomly divided into the control group and the DSS-induced model group, with five mice in each group. Then, the mice in the model group were administered a 3% DSS solution continuously for 7 days, with fresh DSS solution being replaced every 1 day. Meanwhile, the control group was provided with regular drinking water throughout the experimental period.

Daily observations were conducted on the mice, including tracking their condition, recording changes in body weight, and observing cases of bloody stool. On the 8th day, each mouse was euthanized. The serum was collected for ELISA testing, and colonic tissues were harvested for hematoxylin and eosin (H&E) staining, Western blot, and RT-qPCR. The degree of inflammation in the colonic tissue was evaluated through pathological analysis. The concentrations of TNF-α, IL-6, and IL-1β in the serum were precisely measured using ELISA kits (provided by Jianglai Biotechnology Company), following the manufacturer’s recommended protocols.

### 2.14 RT-qPCR analysis

In this study, RT-qPCR was used to assess the expression of biomarkers in tissue samples. A total of 5 pairs of colonic mucosal tissue samples were collected from UC mice induced by DSS and the control group, consisting of 5 UC and 5 control samples. Total RNA from these samples was extracted using TRIzol reagent (Vazyme, Nanjing, China), and RNA concentrations were measured with a NanoPhotometer N50. mRNA was then reverse transcribed into cDNA using a test kit (Yi Sheng, Wuhan, China). RT-qPCR was performed to evaluate the expression of TEX11, SLC16A1, OVOL1, EDNRA, and HEPACAM2. The expression levels of these biomarkers were calculated using the 2^-ΔΔCt^ method, and differences in expression were analyzed using Student’s t-test (*P* < 0.05). Statistical analysis and visualization were carried out using GraphPad Prism 5 (v 8.0) (45). Detailed information on primers and machine testing conditions is provided in **Supplementary Table S2**.

### 2.15 Western blot analysis

Colonic tissue samples were added to RIPA lysis buffer comprising of protein phosphatase inhibitor and homogenized on ice. Following a 30-minute lysis period, the supernatant proteins were collected through centrifugation. The BCA protein quantification kit (Solarbio, Beijing, China) was employed to determine the protein concentration. Subsequently, the 10% SDS-polyacrylamide gel electrophoresis was used to separate 50 μg of total protein, which was then transferred onto a nitrocellulose (NC) membrane. After adding 5% skimmed milk and sealing for 2 hours, the membranes were incubated with primary antibodies (EDNRA antibody, 1:1000, Abcam; SLC16A1, 1:1000, Proteintech) at 4 °C for overnight incubation. On the second day, the secondary antibody (1∶5000) was added and incubated. Later, the image was captured using a gel imaging system. Finally, the Image J image analysis system was employed to conduct an analysis of the grayscale values of the target band.

### 2.16 Recruitment of subjects and sample collection

The study enrolled a total of 5 individuals diagnosed with UC and 5 healthy controls (HCs) who were matched in terms of age and gender. All patients were treated in the gastroenterological department of Henan Provincial Hospital of Traditional Chinese Medicine from June 2025 to July 2025. UC typically manifests with episodes of bloody diarrhea and is diagnosed based on colonoscopy, histopathological analysis. The study protocol was approved by the Ethics Committee of Henan Provincial Hospital of Traditional Chinese Medicine (No. HNSZYYWZ-20250403056).

Following standard bowel cleansing using polyethylene glycol electrolyte solution, participants underwent colonoscopy, during which a single mucosal biopsy was obtained from the colorectal lesion. The specimen was immediately fixed in 10% formalin for subsequent immunohistochemistry analysis.

### 2.17 Immunohistochemistry analysis

After deparaffinization, antigen repair antigen retrieval using EDTA buffer and nonspecific antigen blocking, the sections were incubated with primary antibodies (EDNRA, 1:1000, Abcam; SLC16A1, 1:1000, Proteintech) overnight at 4°C. After being washed extensively with PBS, the slides were incubated with secondary antibody for one hour, followed by visualization with diaminobenzidine. Afterward, the sections were counterstained using hematoxylin. For each tissue section, three distinct non-overlapping fields were randomly chosen and captured at 400× magnification using an OLYMPUS microscope. The acquired images were processed using Image-Pro Plus 6.0 software. The mean optical density (MOD) of the stained mucosal regions was calculated to assess the expression levels of EDNRA and SLC16A1.

### 2.18 Statistical analysis

Bioinformatics analyses were performed using R programming language (v 4.3.1). The Wilcoxon test or Student’s t-test was applied to compare differences between two groups, with *P* < 0.05 considered statistically significant.

## 3 Results

### 3.1 Identification and exploration of candidate genes

**Figure 1:**
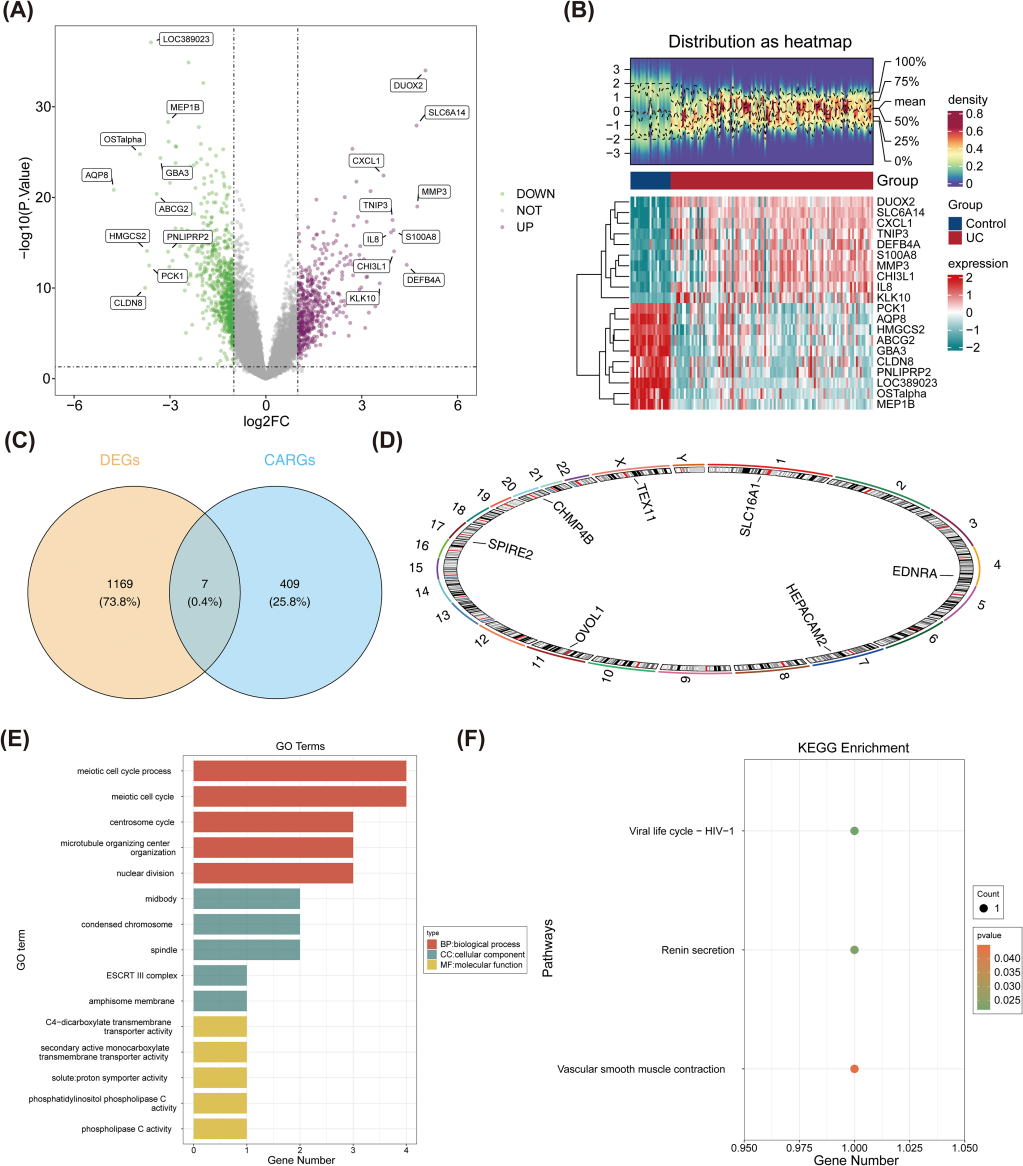
Identification and exploration of candidate genes. Identification of DEGs **(A)**. Volcano plot of DEGs between UC and controls **(B)**. Seven candidate genes obtained through the intersection of DEGs and CARGs **(C)**. Chromosomal location distribution of the candidate genes **(D)**. GO and KEGG enrichment analysis of the candidate genes (**E, F**). DEGs: differentially expressed genes; UC: ulcerative colitis; CARGs: centrosome amplification-related genes.

In GSE87473, 1,176 DEGs were identified, including 487 upregulated and 689 downregulated genes in the UC group (**Figure 1A, B**). The number of DEGs under three different thresholds is shown in **Supplementary Table S1**. It was found that the stringency of the threshold was negatively correlated with the number of DEGs, and loose thresholds significantly increased potential false positives. A total of 795 DEGs were identified under all three thresholds. The screening results using the original threshold included all genes identified under the strict threshold, while avoiding potential noise introduced by loose thresholds (**Supplementary Table S3**, **Supplementary Figure S1**). Seven candidate genes were selected by intersecting DEGs with CARGs (**Figure 1C**). Chromosomal mapping revealed SLC16A1 on chromosome 1, EDNRA on chromosome 4, HEPACAM2 on chromosome 7, OVOL1 on chromosome 11, SPIRE2 on chromosome 16, CHMP4B on chromosome 20, and TEX11 on the X chromosome (**Figure 1D**).

GO analysis identified 255 biological processes (BPs), 22 cellular components (CCs), and 14 molecular functions (MFs) significantly enriched (**Figure 1E**, **Supplementary Table S4**). Candidate genes were primarily enriched in meiotic cell cycle among BPs, midbody and spindle within CCs, and phospholipase C activity in MFs. KEGG pathway analysis further revealed that the candidate genes had potential functional associations in pathways such as renin secretion (**Figure 1F**), which provided clues for understanding their roles in related physiological and pathological processes.

### 3.2 Identification of biomarkers

**Figure 2:**
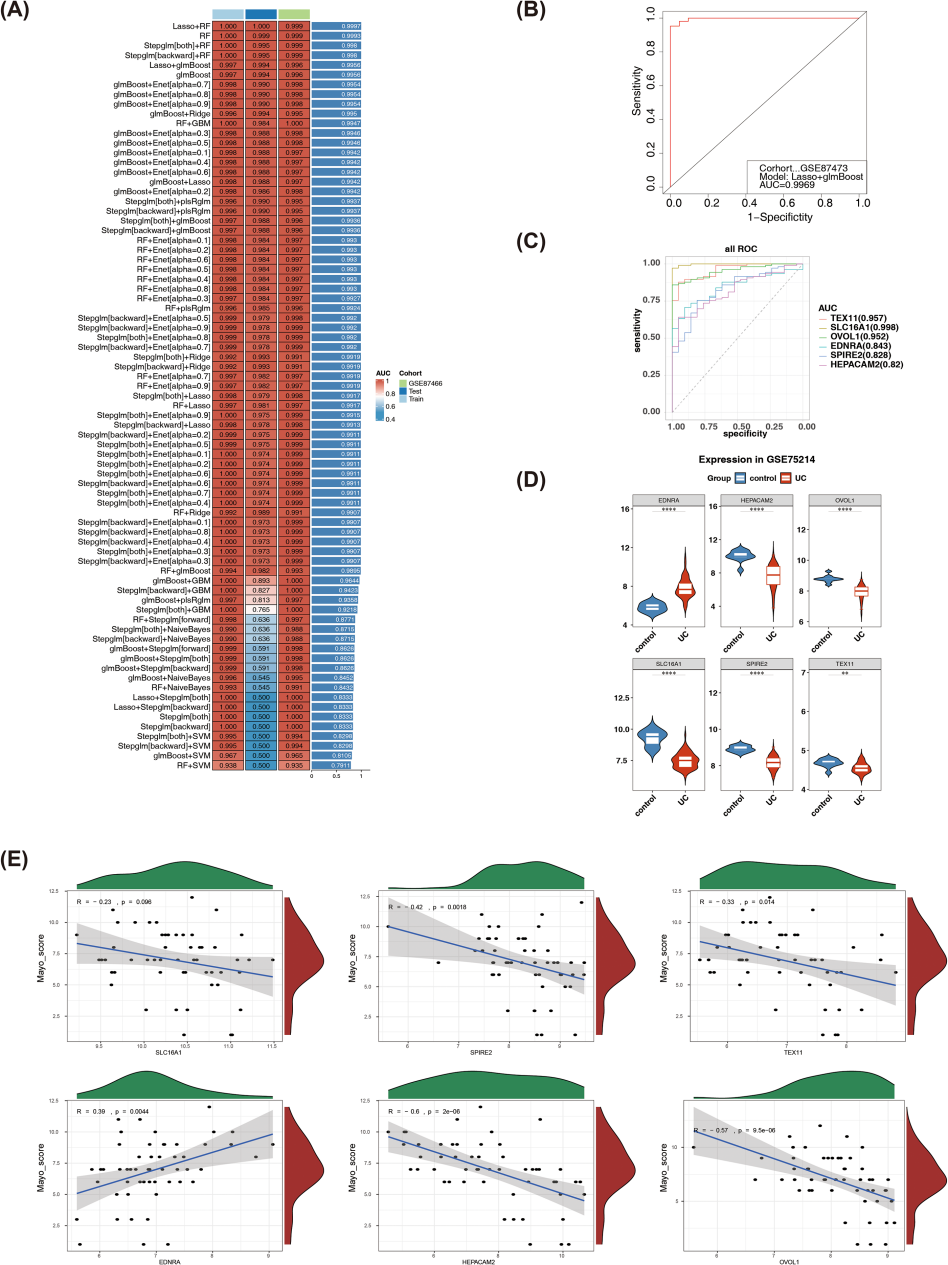
Identification of Biomarkers. Screening of candidate key genes using 101 machine learning algorithms **(A)**. ROC curve evaluating the accuracy of the top-performing algorithm in the training set **(B)**. ROC curves for each gene in the training set **(C)**. Expression levels of candidate key genes in the training set GSE87473 **(D)**. Investigation of the correlation between biomarkers and the Mayo score **(E)**. ROC: Receiver Operating Characteristic curve.

A total of 113 machine learning model combinations were evaluated. The “Lasso + glmBoost” model achieved the highest average AUC. Since models including Lasso+RF, RF, stepglm[both], and stepglm[backward]+RF showed signs of overfitting with AUC values of 1 in both datasets, they were excluded from consideration. The Lasso + glmBoost model was ultimately selected as it demonstrated AUC values of 0.9969 and 0.9939 in the two datasets, respectively, and exhibited the highest mean AUC (**Figures 2A, B**; **Supplementary Figure S2A**). Furthermore, validation in the independent dataset GSE87466 confirmed the model’s robustness, showing an AUC value of 0.9962 (**Supplementary Figure S2B**). Based on these results, six genes (TEX11, SLC16A1, OVOL1, EDNRA, HEPACAM2, and SPIRE2) identified by this model were selected as candidate biomarkers for subsequent analysis.

ROC curve analysis confirmed that each gene achieved AUC values above 0.7 in the two datasets (**Figures 2C**; **Supplementary Figure S2C, D**). Significant differential expression was observed for all six genes between UC and control samples: TEX11, SLC16A1, OVOL1, HEPACAM2, and SPIRE2 were downregulated in UC samples, whereas EDNRA was upregulated (**Figures 2D**; **Supplementary Figure S2E, F**). Consequently, these genes were designated as biomarkers. Correlation analysis revealed a positive association between EDNRA and the Mayo score, while TEX11, OVOL1, HEPACAM2, and SPIRE2 showed significant negative correlations (**Figure 2E**).

SHAP analysis elucidated the specific contribution patterns of each biomarker in UC classification prediction. The feature importance ranking (**Supplementary Figure S3A**) indicated that SLC16A1 (Mean |SHAP| = 0.151) had the highest feature importance, followed by OVOL1 (0.045), TEX11 (0.041), EDNRA (0.022), SPIRE2 (0.007), and HEPACAM2 (0.005). This ranking was entirely consistent with the Random Forest built-in importance analysis (**Supplementary Figure S3B**), confirming SLC16A1 as the most discriminative biomarker. As shown in **Supplementary Figure S3C**, the SHAP beeswarm plot detailed the distribution of SHAP values for each feature. High expression of SLC16A1 (red dots) was predominantly distributed in the positive SHAP value region, indicating that its elevated expression significantly increased UC risk. OVOL1 and TEX11 exhibited similar influence patterns, with high expression positively correlated with UC risk. All six biomarkers displayed clear dose-response relationships, demonstrating good consistency between feature expression levels and SHAP values. These findings indicate that the six genes not only exhibit differential expression but are also associated with disease severity, suggesting their potential roles in the pathogenesis of UC.

### 3.3 Enrichment pathway of biomarkers

GSEA revealed that 90, 80, 86, 67, 87, and 86 pathways were significantly enriched in the gene rankings associated with EDNRA, HEPACAM2, SPIRE2, TEX11, SLC16A1, and OVOL1, respectively (**Supplementary Table S5**) (*P* < 0.05). Notably, the cell adhesion molecule (CAM) pathway was significantly enriched in the gene rankings associated with HEPACAM2, EDNRA, and OVOL1 (**Supplementary Figure S4A-C**), while the oxidative phosphorylation pathway was significantly enriched in the gene rankings associated with HEPACAM2, SPIRE2, TEX11, SLC16A1, and OVOL1 (**Supplementary Figure S4A, C-F**). The above results indicated that the biomarkers might primarily influence the occurrence and progression of UC through these significantly enriched pathways, but the specific regulatory relationships remained to be further verified by experiments.

### 3.4 DICs and DCIFs linked to biomarkers in UC

**Figure 3:**
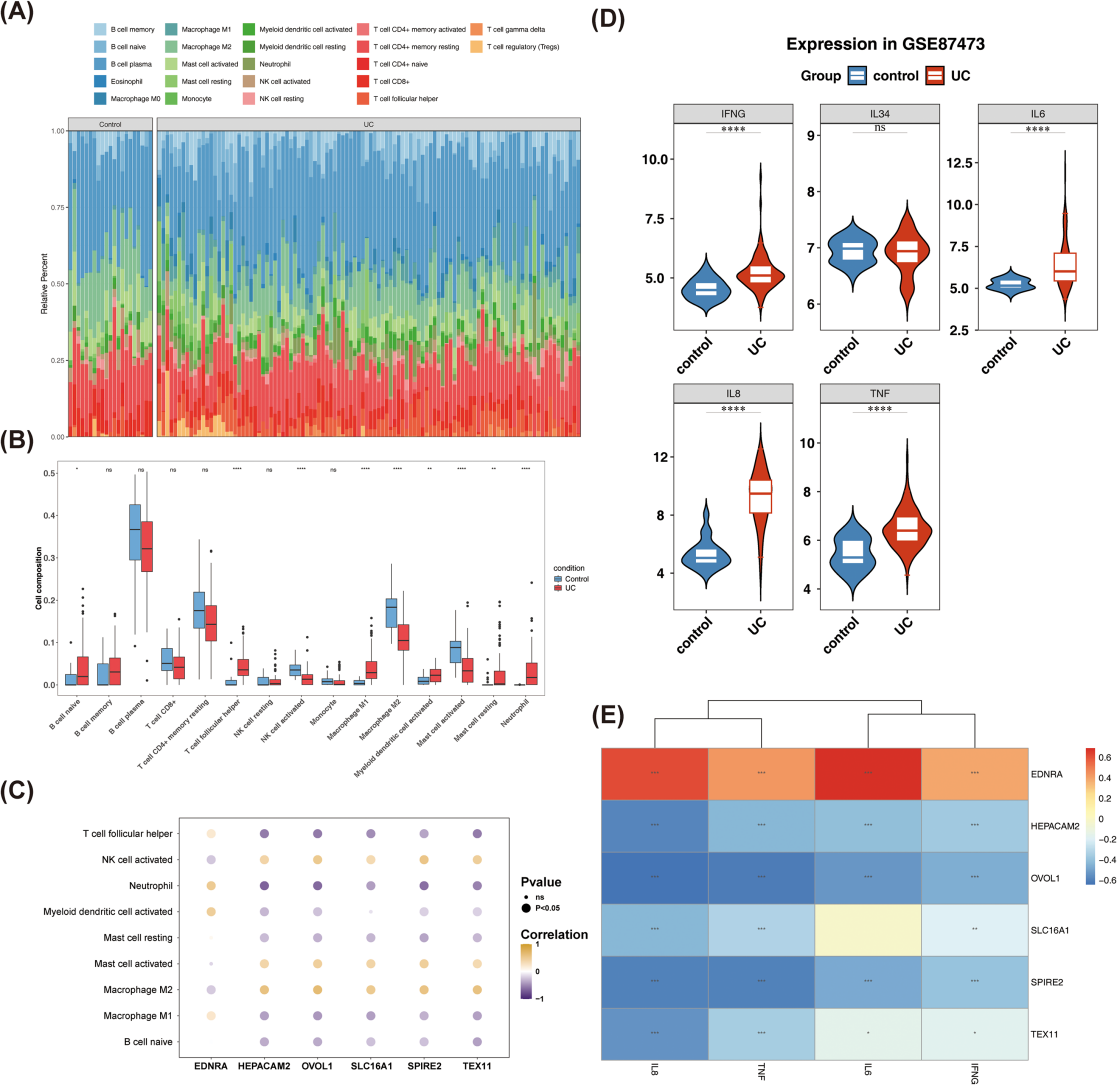
DICs and DCIFs linked to biomarkers in UC. Abundance of 22 immune cell types between UC and controls (A). Nine types of immune cells showed significant differences between UC and controls (B). Correlation analysis of biomarkers with most DICs (C). DCIFs between UC and control samples, including IFNG, IL6, IL8, and TNF, were upregulated in UC (P < 0.05) (D). Correlation analysis of biomarkers with DCIFs (E). DICs: differential immune cells; DCIFs: differential cellular inflammatory factors (*P < 0.05, **P < 0.01 and ****P < 0.0001, ns, No significance).

The abundance of 22 immune cell types was assessed, revealing that plasma B cells constituted the highest proportion in all samples (**Figure 3A**). Seven cells were excluded from analysis due to a lack of infiltration. Wilcoxon test results indicated significant differences in nine immune cell types, including naive B cells, M1 macrophages, and neutrophils, between UC and control samples (**Figure 3B**). Correlation analysis showed significant associations between biomarkers and most DICs. Specifically, neutrophils were negatively correlated with OVOL1, TEX11, SLC16A1, HEPACAM2, and SPIRE2 (*P* < 0.001), while positively correlated with EDNRA (*P* < 0.001) (**Figure 3C**, **Supplementary Table S6**). These results suggest that DICs, such as neutrophils, may interact with biomarkers to influence UC. DCIFs between UC and control samples, including IFNG, IL6, IL8, and TNF, were found to be upregulated in UC (*P* < 0.05) (**Figure 3D**). Correlation analysis revealed a strong positive correlation between DCIFs and EDNRA, while negative correlations were observed with the other five biomarkers. For example, IL8 was negatively correlated with HEPACAM2, OVOL1, SLC16A1, TEX11, and SPIRE2 (R =-0.58, -0.64, -0.43, -0.55, -0.59, *P* < 0.001). IFNG, IL6, IL8, and TNF were positively correlated with EDNRA (R = 0.39, 0.69, 0.61,0.43, *P* < 0.001) (**Figure 3E**, **Supplementary Table S7**). These results suggested that DCIFs may establish a potential functional link between biomarkers and UC, providing clues for a deeper analysis of their regulatory network.

### 3.5 Enrichment pathways and DICs in different clusters

**Figure 4:**
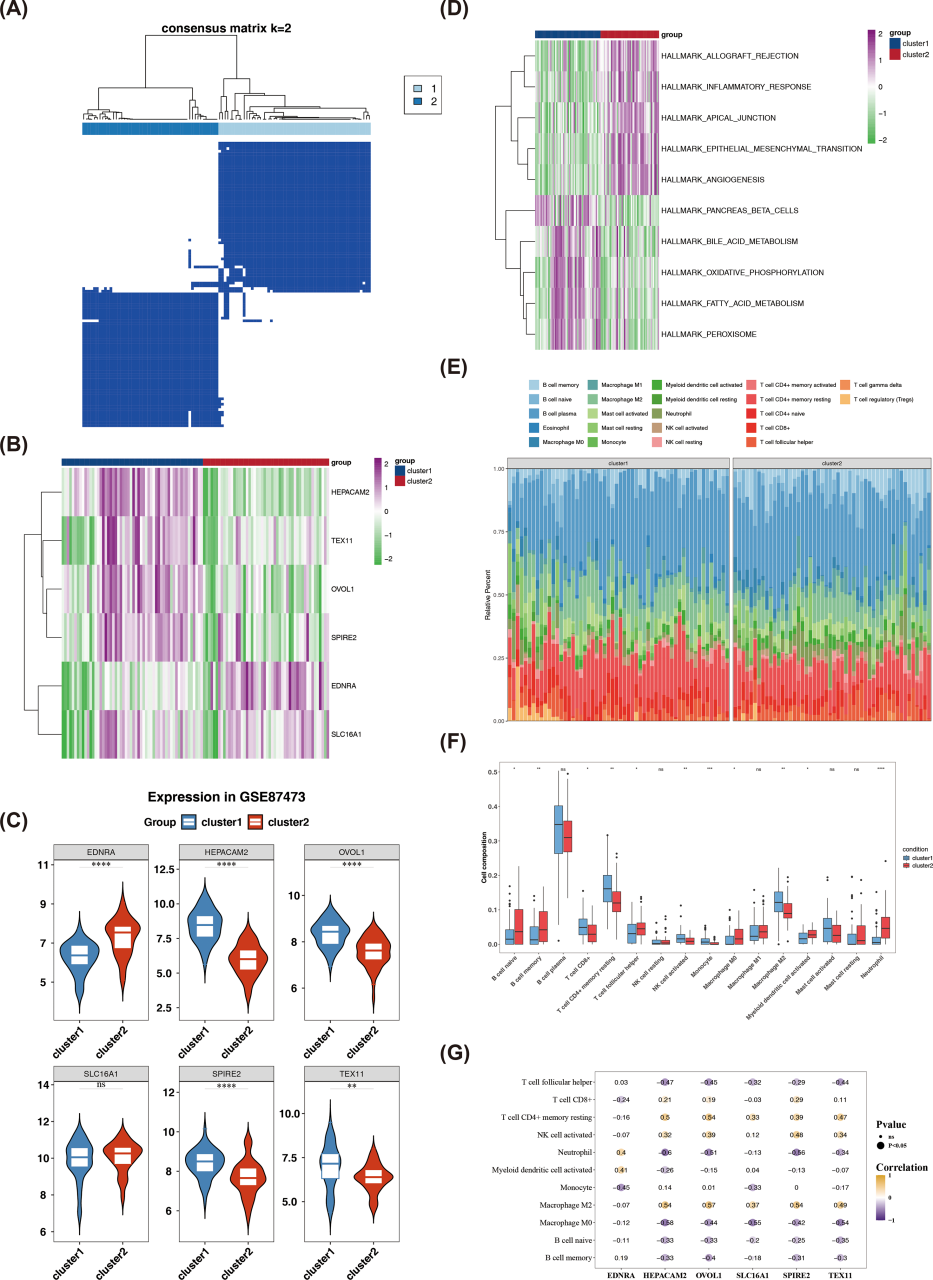
Enrichment pathways and DICs in different clusters. The UC samples in the GSE87473 data were divided into two clusters (clusters 1 and 2) (A). Heatmap of biomarkers in different clusters (B). Box plots of biomarkers in different clusters (C). Differences in enrichment pathways among different clusters were analyzed through GSVA (D). The abundance of 22 immune cell types between clusters 1 and 2 (E). The difference in immune cell infiltration in different clusters (F). Correlation analysis of biomarkers and DICs in different clusters (G). GSVA: gene set variation analysis (*P < 0.05, **P < 0.01,***P < 0.001 and ****P <0.0001, ns, No significance).

As shown in **Figure 4A**, the highest within-community correlation and low intergroup correlations were observed when k **=** 2. Consequently, UC samples from the GSE87473 dataset were divided into two clusters. Expression analysis revealed that TEX11, HEPACAM2, OVOL1, and SPIRE2 were highly expressed in cluster 1, while EDNRA was highly expressed in cluster 2 (**Figure 4B**, **Supplementary Figure S5A**). Wilcoxon test results further indicated that TEX11, OVOL1, HEPACAM2, and SPIRE2 showed downregulation, while EDNRA exhibited upregulation in UC samples from cluster 2 (**Figure 4C**, **Supplementary Figure S5B**). GSVA revealed that pathways such as angiogenesis were suppressed, while pathways related to oxidative phosphorylation were activated in cluster 1 (**Figure 4D**). These results suggest distinct differences in gene expression and pathway enrichment between the two clusters.

The abundance of 22 immune cell types between clusters 1 and 2 is shown in **Figure 4E**. Six cells were excluded from the analysis, and Wilcoxon test results revealed significant differences in 11 immune cell types, including naive B cells and neutrophils, between the clusters (**Figure 4F**). Correlation analysis showed that M0 macrophages were significantly negatively correlated with OVOL1, TEX11, SLC16A1, HEPACAM2, and SPIRE2 (*P* < 0.001) (**Figure 4G**). These results indicated that immune cells, particularly M0 macrophages, may exhibit certain interactions with biomarkers in UC clusters, suggesting their potential role in the underlying mechanisms.

### 3.6 TFs, miRNAs, and chemical compounds related to biomarkers

A total of 131 TFs were predicted from the database, with 10 differential TFs, such as HEF4A and PPARG, identified as linked to biomarkers (**Supplementary Figure S6A, B**). The transcription factor ELK3 was significantly positively correlated with EDNRA (r=0.62, *p* < 0.001), which was consistent with its biological function as an activator. For example, the transcription factor EOMES was significantly negatively correlated with OVOL1 (r=-0.58, *p* < 0.001), in line with its mechanism of inhibiting target gene expression. Therefore, it could be concluded that the expression trends of TFs were highly consistent with the expression patterns of target genes, indicating that transcription factors affected the expression of key genes in UC through direct regulation (**Supplementary Figure S6C**). NANDG was predicted to be associated with SLC16A1, TEX11, OVOL1, and SPIRE2. Additionally, miRNAs such as hsa-miR-335-3p were found to be linked to SLC16A1, HEPACAM2, and EDNRA, while hsa-miR-590-3p was associated with OVOL1 and SLC16A1. hsa-miR-3128 was linked to TEX11, but no miRNAs were found to be associated with SPIRE2 (**Supplementary Figure S6D**). These results suggest that these factors, linked to biomarkers, may play pivotal roles in UC progression. Finally, chemical compounds such as valproic acid were identified as associated with EDNRA and OVOL1, while bisphenol A was linked to TEX11, SLC16A1, OVOL1, SPIRE2, and HEPACAM2 (**Supplementary Figure S6E**). These findings highlight that these chemical compounds, associated with multiple biomarkers, could potentially influence UC pathology in patients.

### 3.7 The expression of biomarkers in cells

**Figure 5:**
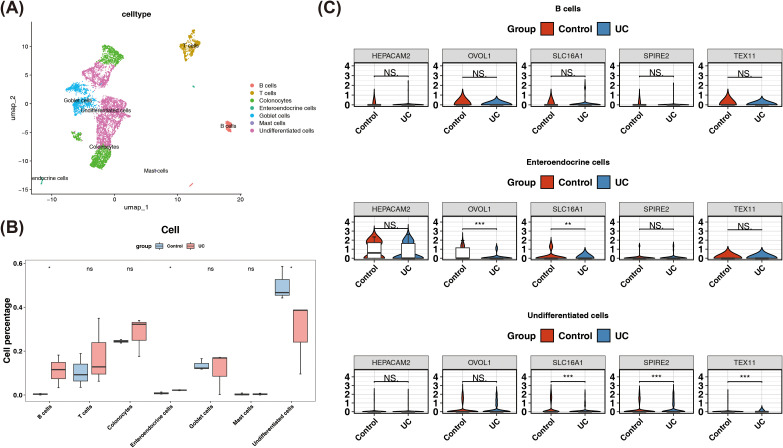
Single-cell Analysis. Results of cell annotation analysis (A). Cell type differences between the ulcerative colitis (UC) group and the control group, and the expression of marker genes in different cell types (B). Expression of key genes in differentially expressed cells (C) (*P < 0.05, **P < 0.01 and ***P < 0.001, Ns, No significance).

After quality control, 7,005 cells and 21,256 genes were retained for further analysis (**Supplementary Figure S7A, B**). The top 2,000 hypervariable genes were selected for PCA (**Supplementary Figure S7C-E**). Subsequently, the top 30 principal components were chosen for clustering, resulting in the division of all cells into 15 clusters (**Supplementary Figure S7F**). Marker gene expression analysis revealed that genes such as CD79A and TPSAB1 were highly expressed in specific cell types (**Supplementary Figure S7G**). A total of seven cell types, such as B cells, T cells, enteroendocrine cells (EECs), and undifferentiated cells, were annotated based on marker gene expression within these clusters (**Figure 5A**). The proportion of B cells and EECs was significantly higher in the UC group, while undifferentiated cells were more prevalent in the control group (**Figure 5B**). These three cell types were thus classified as differential cells. Further analysis revealed that OVOL1 and SLC16A1 showed significant expression differences in EECs, whereas SPIRE2, TEX11, and SLC16A1 exhibited notable differences in undifferentiated cells. EDNRA expression was undetectable, and HEPACAM2 showed no significant expression across any cell types (**Figure 5C**). These results suggested that undifferentiated cells and EECs may play a potential role in UC research, meriting further investigation into their relevance.

**Figure 6:**
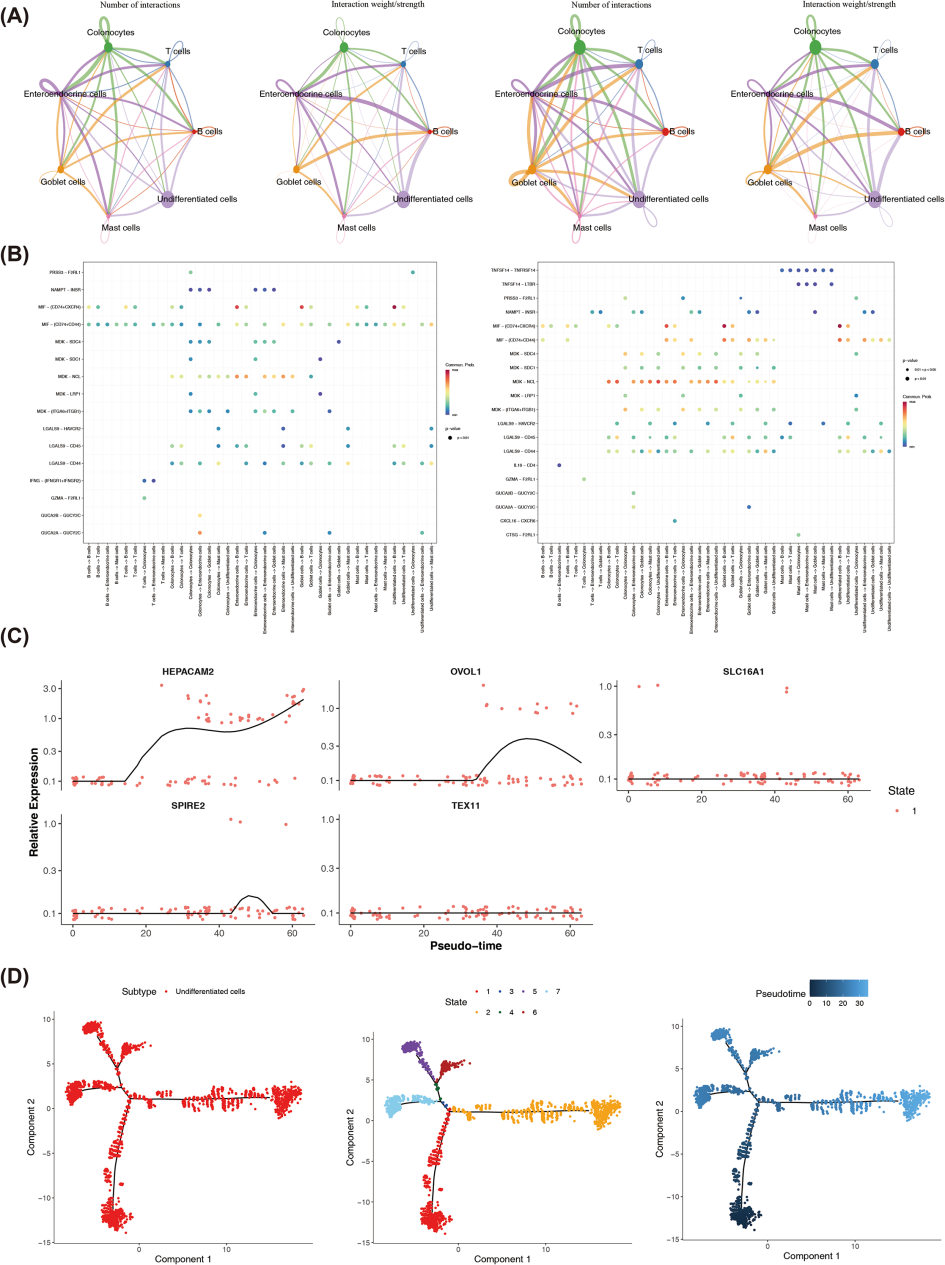
Cell Communication Analysis and Pseudotime Analysis. Number and strength of cell communications in the control group versus the UC group **(A)**. Communication networks between cells in the control group versus the UC group **(B)**. Expression changes of key genes over pseudotime **(C)**. Cell differentiation trajectory **(D)**.

Cell communication analysis showed that in the UC group, both the number and weight of cell communications were increased; for example, enteroendocrine cells and undifferentiated cells had strong communication relationships with other cells (**Figure 6A**). In control and UC groups, relatively high communication probabilities were observed between undifferentiated cells and B cells, with the ligand-receptor pair being MIF- (CD74+CXCR4) (**Figure 6B**). This discovery provided potential clues for further analysis of the cellular interaction network and its regulatory mechanisms within the UC microenvironment.

Pseudo-time analysis showed that the differentiation trajectories of enteroendocrine cells developed from the dark blue to the light blue. Enteroendocrine cells persisted throughout the entire cell differentiation stage. The cell development stages were divided into 1 period (**Supplementary Figure S8A**). HEPACAM2 showed a gradually increasing trend over time, OVOL1 exhibited a trend of first increasing and then decreasing, and SPIRE2 displayed a trend of first increasing, then decreasing, and subsequently leveling off (**Figure 6C**).

Pseudo-time analysis showed that the differentiation trajectories of undifferentiated cells developed from the dark blue to the light blue. Undifferentiated cells persisted throughout the entire cell differentiation stage. The cell development stages were divided into 7 periods (**Figure 6D**). SLC16A1 showed a trend of first decreasing and then leveling off over time, while SPIRE2 exhibited a gradually decreasing trend (**Supplementary Figure S8B**). These temporal expression patterns suggested that the aforementioned genes may play stage-specific roles in cellular regulation, and their dynamic changes provided potential clues for understanding the evolution of UC-related cellular functions.

### 3.8 Validation of biomarkers expression in the mouse model of UC

**Figure 7:**
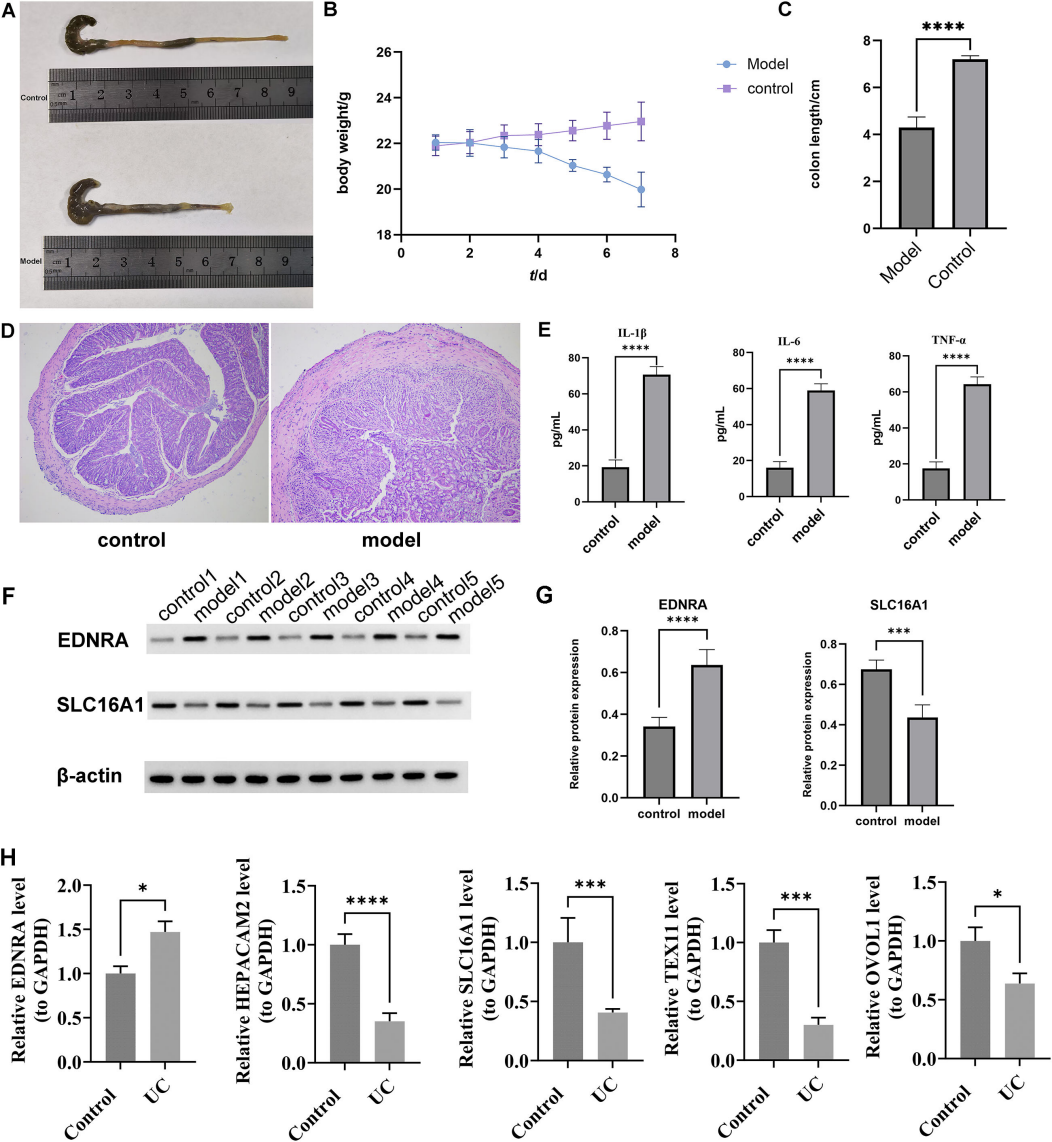
Validation of biomarkers expression in the mouse model of UC. Colon length and body weight of different groups of mice (A-C). Representative HEstaining of different groups (Original magnification: 100×) (D). Serum inflammatory cytokines detection of different groups (E). Western blot analysisof differential protein expression results (F, G). RT-PCR analysis of differential gene expression results (H). The asterisks denote the statisticalsignificance levels (*P < 0.05, ***P < 0.001, and ****P < 0.0001).

To validate the expression of biomarkers in UC, we evaluated the colon tissues and serum of mice. The model group exhibited significant differences in colon length and body weight when compared to the control group (**Figures 7A-C**). We conducted HE staining and serum inflammatory factor detection to confirm the successful establishment of the mouse model of UC, as shown in **Figures 7D, E**. The levels of IL-1β, TNF-α, and IL-6 were significantly higher in UC (*P* < 0.0001).

In Western blot experiments, the expression of EDNRA in mouse intestinal inflammatory tissues was significantly higher in UC patients than that in controls. The expression of SLC16A1 in colonic mucosa specimens decreased significantly in UC model group, as depicted in **Figures 7F, G**.

In RT-qPCR experiments using colonic mucosal tissues of mice, expression levels of five biomarkers showed significant differences between UC and control samples (*P* < 0.05). Specifically, SLC16A1, OVOL1, TEX11, and HEPACAM2 were downregulated in UC samples, while EDNRA was upregulated compared to control samples (**Figure 7H**). These results visually indicated that biomarkers exhibited differential expression in the UC mouse model, which might be associated with the pathogenesis and progression of the disease, warranting further investigation into their potential biological significance.

### 3.9 Validation of biomarkers expression in the UC patients and HCs

The study included 5 patients with UC and 5 age - and sex - matched HCs. The male-to-female ratio was 3:2 in the UC group versus 2:3 in the HC group (*P* = 0.527), and the average age was 44.4 ± 6.35 years in the UC patient group compared to 49.6 ± 10.78 years in the HC group. (*P* = 0.38).

**Figure 8:**
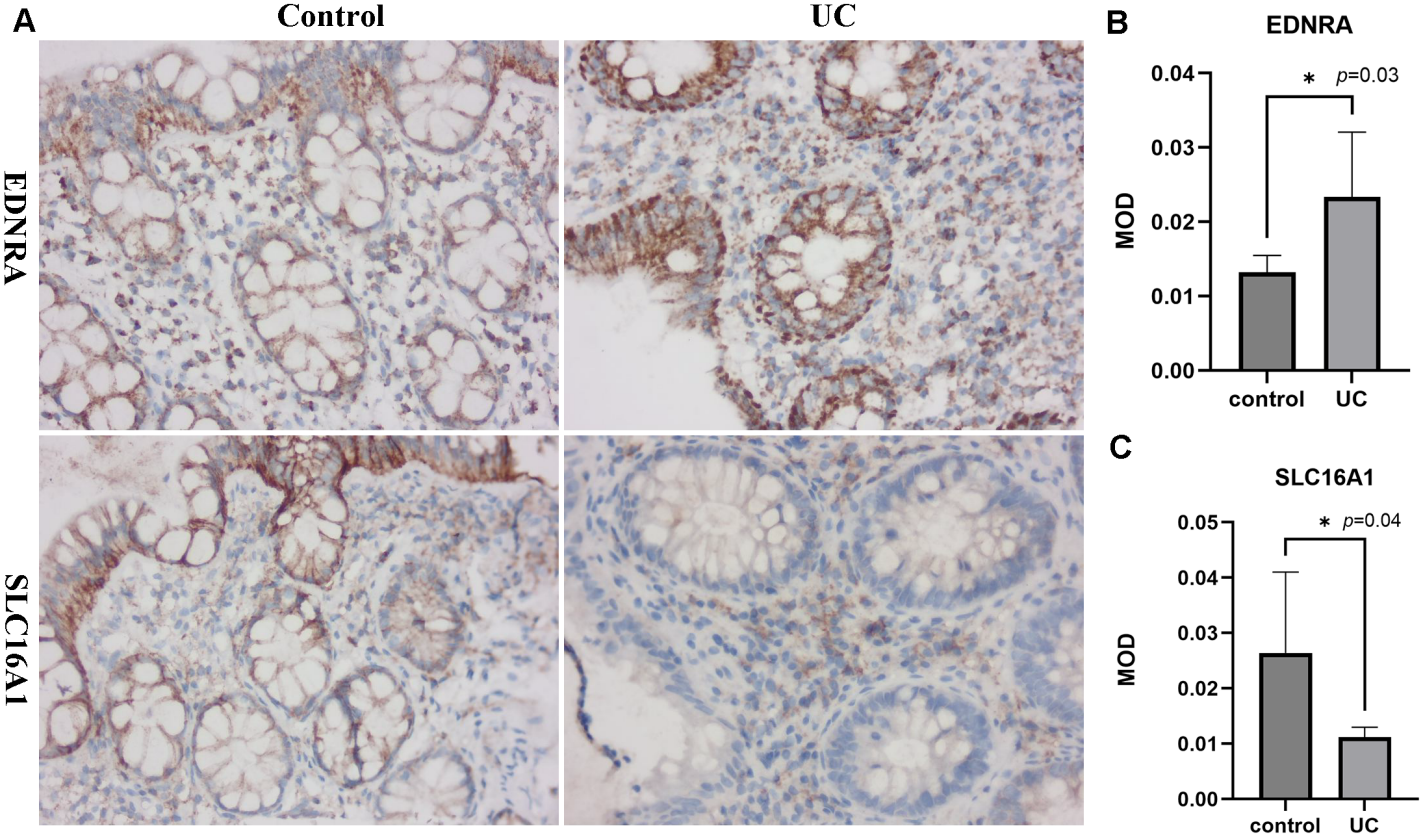
Mucosal immunohistochemistry in patients with UC and HCs. Representative photomicrographs of the immunoreactivity of EDNRA and SLC16A1 in UC patients and HCs (Original magnification: 400×) (A). The mean optical density of EDNRA and SLC16A1in the colonic mucosa of UC patients and HCs, *p < 0.05 (B, C).

**Figure 8A** illustrated the expression of EDNRA and SLC16A1 in the colonic mucosa of patients with UC and HCs in immunohistochemistry experiments (×400 magnification). In both UC patients and HCs, EDNRA immunoreactivity was observed in the epithelial layer and lamina propria. In contrast, SLC16A1 staining in HCs was predominantly localized to the epithelium. In patients with UC, the MOD of EDNRA in the colonic mucosa was significantly higher compared to that observed in HCs (*P* = 0.03) (**Figure 8B**). Conversely, the MOD of SLC16A1 in colonic mucosal specimens was significantly reduced in patients (*P* = 0.04) (**Figure 8C**). These results demonstrated that the expression changes and localization characteristics of EDNRA and SLC16A1 in clinical samples further supported their potential role in disease development.

## 4 Discussion

The exact mechanisms underlying the formation of UC remain unclear. In recent years, RNA-seq has emerged as a highly efficient method for studying disease development and identifying molecular abnormalities (46). Additionally, integrative bioinformatics analysis combined with machine learning techniques is increasingly used to uncover biomarkers, underlying mechanisms, and potential therapeutic targets (47, 48). This study systematically uncovers the role of CA-related genes in UC by integrating these computational methods with experimental validation, offering a novel perspective for comprehending the pathological mechanisms of UC.

Current research has not fully elucidated the direct mechanism of action of CA in UC. However, based on the available data, CA may influence UC by regulating the regeneration of intestinal epithelial cells, barrier function, and overall intestinal homeostasis. The centrosomal protein FGFR1OP deletion in mouse intestinal epithelial cells disrupted crypt architecture, impairing stem cell division and reducing intestinal epithelial renewal capacity. The findings also indicated that FGFR1OP was essential for preserving the cell cytoskeleton and cell–cell adhesion within intestinal crypts (49). Remarkably, extra centrosomes induce death-domain protein 1 (PIDD1)-mediated inflammation and immunosurveillance. PIDD1 promotes NF-κB signaling upon cytokinesis failure (50). Therefore, restoring the normal function of the centrosome and maintaining the stability of the intestinal epithelium may be a promising therapeutic strategy for UC worth exploring in the future.

In this study, differentially expressed CARGs were explored across two UC datasets. Machine learning algorithms, ROC analysis, and expression validation were applied to identify biomarkers. Six biomarkers—TEX11, SLC16A1, OVOL1, EDNRA, HEPACAM2, and SPIRE2—were found to have potential associations with UC. These biomarkers were associated with cell adhesion molecules and oxidative phosphorylation pathways. Their expressions were correlated with the infiltration levels of immune cells such as neutrophils. Consistency clustering divided UC samples into two clusters, with DICs, including M0 macrophages, showing notable correlations with biomarkers. Single-cell level analysis highlighted that the expression differences of biomarkers between undifferentiated cells and EECs were the most pronounced. RT-qPCR analysis, Western blot, and Immunohistochemistry analysis confirmed that SLC16A1, OVOL1, TEX11, and HEPACAM2 were downregulated in UC samples compared to controls, while EDNRA was upregulated, aligning with bioinformatics predictions.

The testis-expressed 11 (TEX11), located on the X chromosome, plays a critical role in spermatogenesis (51) and has been confirmed as an important factor in maintaining genomic stability (52). TEX11 has been regarded as a potential biomarker for early-onset colorectal cancer (CRC) based on database analyses. It shows significantly under-expressed in CRC and is correlated with poor prognosis in patients (53). Furthermore, TEX11 is involved in pubertal and reproductive deficiencies in humans (54). Notably, we find that the expression of TEX11 was also significantly downregulated in UC samples. Further mechanistic exploration reveals that a positive correlation between TEX11 expression and the oxidative phosphorylation pathway. These findings are consistent with previous studies demonstrating that the upregulation of TEX11 mediates inflammation and oxidative stress through the HDAC4-FoxO3a axis (55). Oxidative phosphorylation, as a core process in cellular energy metabolism, its dysfunction can lead to excessive production of ROS, thereby inducing oxidative stress (56). Both genomic instability and oxidative stress are well-known mechanisms that induce CA (57). Therefore, the findings of this study suggest that downregulation of TEX11 expression may be involved in centrosome dysfunction in UC through interrelated pathways: impaired cellular energy metabolism and genomic instability. This discovery provides novel insights into the role of TEX11 in the pathogenesis of UC. Future studies will aim to elucidate the specific regulatory mechanisms of TEX11 in the oxidative phosphorylation pathway and its causal relationship with centrosome stability.

Solute carrier family 16 member 1 (SLC16A1), commonly referred to as MCT1, is a key membrane transport protein responsible for facilitating the transmembrane transport of lactate and pyruvate (58). Research further supports the idea that MCT1 plays an important biological role in macrophages (59). SLC16A1 expression in macrophages enables them to absorb lactate, promoting their differentiation into a regulatory anti-inflammatory phenotype, commonly referred to as the M2 phenotype (60). This study demonstrates that SLC16A1 expression is downregulated in UC tissues, and immune infiltration analysis reveals an increase in pro-inflammatory M1 macrophages, which aligns with prior findings (61, 62). It should be noted that the lactate transport mediated by SLC16A1 is crucial for maintaining cellular metabolic homeostasis, and the cellular metabolic state directly influences the energy and biosynthetic resources required for CA (63). In the context of UC, downregulated SLC16A1 expression may lead to lactate metabolism disorders, which not only affects energy supply and mucosal regeneration in intestinal epithelial cells (64, 65) but may also indirectly impact centrosome function through disruption of cellular metabolic homeostasis. These findings indicate that dysregulation of SLC16A1 expression is associated with metabolic disturbances and immune imbalance in UC, potentially involving the regulation of centrosome stability.

The TF OVOL1, encoded by the ovo-like 1 gene, is a vertebrate homolog of Drosophila OVO. OVOL1 is expressed in various epithelial cells (66). OVOL1 plays a critical role in maintaining differentiated epidermal cells (67). Additionally, OVOL1 regulates the stemness of cancer cells, significantly contributing to cancer cell metastasis (68). OVOL1 regulates epidermal barrier integrity and neutrophil accumulation in psoriasis-like inflammation (69, 70). Our research has revealed that OVOL1 exhibits significantly low expression in UC and is associated with the cell adhesion molecule pathway. Considering that OVOL1 is a key regulatory factor in epithelial differentiation (71) and that centrosome positioning is involved in the process of epithelial morphogenesis (72), research results suggest that the downregulation of OVOL1 expression may affect the adhesion function of epithelial cells, impairing the stability of centrosomes and thereby participating in the progression of UC. This potential mechanism warrants further research and verification.

Endothelin receptor type A (EDNRA) is a key marker for pericytes and is commonly found on smooth muscle cells (SMCs) that line the vasculature. In patients with high-risk multiple myeloma, EDNRA expression is significantly higher compared to those with low-risk MM, with the highest expression observed in focal lesions (73). The EDN1/EDNRA/β-arrestin pathway promotes CRC progression through the modulation of STAT3 phosphorylation (74). EDNRA also plays a role in the susceptibility to large artery atherosclerotic stroke, possibly through inflammatory mechanisms (75). Our research has found that EDNRA is the only biomarker significantly upregulated among six biomarkers in UC. Its expression level is positively correlated with the Mayo score and pro-inflammatory factors IL-6 and TNF, suggesting that high expression of EDNRA is associated with the degree of inflammation in UC. Current research indicates that the activation of EDNRA can stimulate the STAT3 signaling pathway, and the abnormal activation of the STAT3 signaling pathway is known to regulate CA (76). Based on these evidences, the upregulation of EDNRA in UC may participate in the regulation of centrosome stability by activating the STAT3-related signaling pathway and form a mutually reinforcing vicious cycle with local inflammatory responses. In the future, we can conduct verification from this direction.

Hepatocyte adhesion molecule 2 (HEPACAM2), as a member of the immunoglobulin-like superfamily, exhibits significant regulatory effects in various diseases. Research indicates that HEPACAM2 is highly expressed in small cell lung cancer (77). HEPACAM2 was initially identified as one of three genes located in a hotspot region on chromosome 7q (78). Its expression is elevated in adenomas, correlating with cell-cell adhesion and contributing to tumor metastasis (79). In patients with CRC, decreased HEPACAM2 expression is associated with poor overall survival OS (80). HEPACAM2 can function as a promising biomarker for different subtypes of UC, offering promising avenues for targeted molecular treatments and immunotherapies for UC (81). These research findings suggest that HEPACAM2 may hold significant pathophysiological implications in digestive tract diseases. Notably, this study has identified a significant downregulation of HEPACAM2 expression in UC, and its expression level is significantly correlated with both oxidative phosphorylation and cell adhesion pathways. Mechanistically, as an important cell adhesion molecule, HEPACAM2 can mediate the inter-cellular adhesion process through homotypic or heterotypic interactions and co-localize with the cytoskeleton system (82). Considering that the cytoskeletal network plays a crucial role in coordinating centrosome functions (83), the results of this study suggest that the abnormal expression of HEPACAM2 in UC may interfere with the normal regulation of centrosomes by the cytoskeleton through affecting the adhesion function of intestinal epithelial cells. Moreover, the intrinsic connection between HEPACAM2 and the oxidative phosphorylation pathway further indicates that HEPACAM2 may be involved in multiple biological processes such as energy metabolism regulation and cellular structural stability in the pathogenesis of UC. These findings provide new research directions for in-depth exploration of the molecular mechanisms of UC.

SPIRE2, also known as the nucleating factor for F-actin, plays a critical role in long-distance vesicle transport. Reduced expression of SPIRE2 has been associated with epilepsy (84). In the field of tumor research, SPIRE2 has been proven to interact with miR-195 through the ceRNA mechanism, thereby regulating fatty acid synthase (FASN) and playing a significant role in malignant meningiomas (85). From the perspective of molecular mechanisms, SPIRE2 directly influences the processes of vesicle transport and cytoskeleton reorganization by participating in the regulation of actin dynamics (86). These cellular activities are accomplished through the centrosome-mediated microtubule network (87). This study reveals that the dysregulation of SPIRE2 expression may interfere with the normal operation of the vesicle transport system, disrupt the material exchange between the centrosome and other intracellular compartments, and thus contribute to the disease progression of UC.

This study found that there is significant remodeling of the immune microenvironment in patients with UC and identified nine immune cell types, including naive B cells, M1 macrophages, and neutrophils, that showed significant differences between UC and control samples. Correlation analysis revealed that neutrophils were negatively correlated with OVOL1, TEX11, SLC16A1, HEPACAM2, and SPIRE2, while positively correlated with EDNRA. These results suggest a potential association between neutrophil infiltration and the dysregulated expression of genes related to CA. Relevant research indicates that during the active phase of UC, macrophages in the intestinal microenvironment predominantly polarize into the pro-inflammatory M1 phenotype (88),which degrades tight junction proteins, disrupts the epithelial barrier, and contributes to excessive inflammation (89). Conversely, M2 phenotype macrophages with tissue repair functions are relatively insufficient in UC. Meanwhile, as key effector cells in the inflammatory response, neutrophils infiltrate the intestinal mucosa in UC in substantial numbers. They release serine proteases, which cause direct tissue injury and contribute to the formation of characteristic crypt abscesses (90). Interleukin-22 regulates neutrophil recruitment in UC and is linked to resistance to ustekinumab treatment (91). Based on the aforementioned immune characteristics, this study classified patients with UC into two distinct subgroups through consensus clustering. Among them, patients in cluster 1 exhibited a high-expression pattern of CA-related genes such as TEX11, HEPACAM2, OVOL1, and SPIRE2, accompanied by unique immune cell composition and pathway activation characteristics. These findings not only reveal the heterogeneity existing in UC patients but also provide insight into the molecular mechanisms underlying different UC subgroups. The differences in immune cell distribution and signaling pathways (such as the angiogenesis pathway) between different subgroups further indicate that the immune microenvironment and centrosome function may jointly contribute to shaping the disease phenotype of UC, which offers a theoretical basis for the development of targeted treatment strategies for specific UC subgroups.

This study, through bioinformatics analysis, has identified that ten differentially expressed TFs exhibit a significant association with CA-related biomarkers in UC. Among these are key regulatory factors, such as HNF4A and PPARG, which are already recognized for their involvement in the pathogenesis of UC. Correlation analysis reveals a positive correlation between the transcription factor ELK3 and the expression of EDNRA, which is consistent with the reported function of ELK3 as a transcriptional activator (92). Meanwhile, HNF4A and PPARG are significantly positively correlated with SLC16A1, TEX11, OVOL1, HEPACAM2. The significant roles of these TFs in UC have been supported by extensive studies. PPARG belongs to a family of nuclear receptors and is known to play key roles in regulating metabolism, controlling inflammation and modulating immune processes (93, 94). Patients with UC have down-regulated PPARG gene expression (95, 96). The study has indicated that PPARG suppresses M1 macrophage polarization and the activation of the NLRP3 inflammasome (97). Similarly, a reduction in the expression of HNF4A is also associated with the onset of UC (98, 99). Animal experiments have shown that the absence of HNF4α increases the susceptibility to colitis (100) and genetic studies have also confirmed its crucial role in maintaining the intestinal barrier function (101–103). Additionally, this study has identified multiple miRNAs associated with biomarkers. Hsa-miR-335-3p is associated with SLC16A1, HEPACAM2, and EDNRA, while miR-590-3p is associated with OVOL1 and SLC16A1, and miR-3128 is associated with TEX11. MicroRNAs are small non-coding RNAs that can regulate gene activity and participate in numerous essential biological processes like proliferation, differentiation, and other physiological functions (104, 105). Bioinformatics analysis revealed that miR-335 modulates the WNT and TGFβ signaling pathways (106). The expression of miR-590 was found to be lower in the patients of UC compared to controls (107). Exosomal miR-590-3p derived from M2 macrophages alleviates inflammatory responses and enhances epithelial tissue repair (108). These findings suggest that TFs and miRNAs may jointly form a complex regulatory network. By influencing the expression of CA-related genes, they participate in the inflammatory response, immune regulation, and maintenance of intestinal barrier function in UC. Among them, relevant oxidative phosphorylation signaling pathways may be crucial components of this regulatory network, which warrants further investigation.

The proportion of B cells and EECs was significantly higher in the UC group, while undifferentiated cells were more prevalent in the control group. Expression analysis revealed significant differences in OVOL1 and SLC16A1 in EECs, and SPIRE2, TEX11, and SLC16A1 in undifferentiated cells. These findings suggest that undifferentiated and EECs may be particularly relevant for further study in UC. EECs are chemosensory cells within the intestinal epithelium (109) that produce various chemical messengers involved in gastrointestinal motility, secretion, absorption, and responses to food intake (110). EECs, constituting approximately 1% of the intestinal epithelium, are increasingly recognized as crucial sensors for gut microbiota and microbial metabolites. They are essential in regulating mucosal innate immunity, gut barrier integrity, and visceral sensitivity, all of which influence the progression of gastrointestinal diseases, including IBD (111). EECs are specialized hormone-secreting cells in the intestine. Their differentiation is regulated by key signaling pathways such as Wnt, Notch, and MAPK (112, 113). In this study, HEPACAM2 showed a gradually increasing trend over time, which may affect the differentiation microenvironment of EECs by regulating the adhesion properties of intestinal epithelial cells as a cell adhesion molecule.

This study identified six biomarkers—TEX11, SLC16A1, OVOL1, EDNRA, HEPACAM2, and SPIRE2—related to CA in UC through bioinformatics analysis of public datasets. Using methods such as GSEA, immune infiltration analysis, consistent clustering, TF and miRNA prediction, and scRNA-seq analysis, the potential molecular mechanisms of CARGs as biomarkers for UC were explored. Upstream regulatory factors associated with these biomarkers were also identified, and the biomarkers’ cellular expression was investigated. However, there are limitations to this study. Firstly, as a computational biology study, the analysis primarily relies on retrospective data from public datasets, which may introduce case selection bias and limit the generalizability of the findings. Secondly, although we have established an association between biomarkers and CA through bioinformatics methods, the direct link between genes and CA still requires experimental validation. Additionally, the relatively small sample size of animal and clinical validation in this study may affect the robustness of the statistical conclusions. Finally, direct experimental evidence is still needed to support the mechanistic connection between these genes and the pathogenesis of UC. In future research, gene manipulation experiments (such as gene knockout/overexpression in human colonic epithelial cells or organoids) will be conducted to directly verify the effects of these biomarkers on centrosome morphology and function. The diagnostic value of these biomarkers will be validated in a clinical cohort with an enlarged sample size. Meanwhile, high-resolution microscopy techniques will be used to observe the dynamic changes of centrosomes in UC models to establish a causal relationship between centrosomes and inflammatory signal transduction.

## Data Availability

The datasets analyzed for this study can be found in the[Gene Expression Omnibus (GEO) database [http://www.ncbi.nlm.nih.gov/geo/, GSE87473, GSE75214, GSE92415, GSE87466, and GSE116222]. The original contributions presented in the study are included in the article. Further inquiries can be directed to the corresponding author.

## Supplementary material

Supplementary Figure 1 The Venn diagram of DEGs through the intersection of three thresholds.

Supplementary Figure 2 Identification of Biomarkers. (**A-B**) ROC curves evaluating the accuracy of the top-performing algorithm in GSE75214 (**A**) and GSE87466 (**B**). (**C-D**) ROC curves for each gene in the validation sets GSE75214 (**C**) and GSE87466 (**D**). (**E-F**) Expression levels of candidate key genes in the validation sets GSE75214 (**E**) and GSE87466 (**F**) ROC: Receiver Operating Characteristic curve.

Supplementary Figure 3 SHAP Analysis (**A**) HAP feature importance plot. The x-axis represents the mean absolute SHAP value, and the y-axis lists biomarkers in descending order of importance. (**B**) Random forest built-in importance analysis. (**C**) HAP beeswarm plot. The x-axis represents the SHAP value, and the y-axis similarly shows features sorted by importance. The color of the points indicates feature expression levels (red for high expression, purple for low expression).

Supplementary Figure 4 Enrichment pathway of biomarkers. The top 5 pathways significantly enriched in biomarkers according to GSEA (**A-F**). The cell adhesion molecule (CAM) pathway was significantly enriched in the gene rankings associated with HEPACAM2, EDNRA, and OVOL1 (**A-C**), while the oxidative phosphorylation pathway was significantly enriched in the gene rankings associated with HEPACAM2, SPIRE2, TEX11, SLC16A1, and OVOL1 (**A, C-F**)

Supplementary Figure 5 Cluster Analysis and Expression of Key Genes in Different Subtypes. (**A**) Heatmap distribution of biomarkers in different clusters. (**B**) Boxplot of biomarker expression in different clusters.

Supplementary Figure 6 TFs, miRNAs, and chemical compounds related to biomarkers. Ten differential transcription factors (TFs) were obtained through the intersection of DEGs and TFs (**A**). TFs - Key Genes Regulatory Network (**B**). Regulatory relationships between TFs and biomarkers (**C**). miRNAs - Key Gene Regulatory Network (**D**). Chemical Compounds - Key Gene Relationship Network (**E**).

Supplementary Figure 7 Single-cell Analysis Data Processing and Quality Control. (**A-B**) Violin plots showing the distribution of nFeature_RNA, nCount_RNA, and percent.mt for sample and control samples before (**A**) and after (**B**) quality control. (**C**) Selection of highly variable genes. (**D**) JackStraw plot for Principal Component Analysis. (**E**) Elbow plot for Principal Component Analysis. (**F)** UMAP algorithm for dimensionality reduction and clustering visualization of cell clusters. (**G**) Bubble plot showing the expression of 7 marker genes in different cell types.

Supplementary Figure 8 Cell Communication Analysis and Pseudotime Analysis. (**A**) Differentiation trajectory of enteroendocrine cells. (**B**) Expression of key genes at different differentiation stages.

Supplementary Table 1 The 416 CARGs were obtained by searching for “centrosome amplification” in the Gene Ontology (GO, http://geneontology.org/) and Kyoto Encyclopedia of Genes and Genomes (KEGG, https://www.kegg.jp/).

Supplementary Table 2 The detailed information of primers and machine testing conditions was listed in Supplementary Table S2 in RT-qPCR analysis.

Supplementary Table 3 The number of genes that are upregulated and downregulated at different thresholds.

Supplementary Table 4 In GO analysis, a total of 255 biological processes (BPs), 22 cellular components (CCs), and 14 molecular functions (MFs) were obtained.

Supplementary Table 5 GSEA results showed that the numbers of notable pathways enriched by EDNRA, HEPACAM2, SPIRE2, TEX11, SLC16A1 and OVOL1 were 90, 80, 86, 67, 87 and 86, respectively.

Supplementary Table 6 The correlation analysis demonstrated that biomarkers were notably correlated with most differential immune cells (DICs).

Supplementary Table 7 The correlation analysis demonstrated that DCIFs were notably optimistically correlated with EDNRA and DCIFs were unfavorably correlated with other 5 biomarkers.
