# Supplementary figures and images for: Identification and validation of biomarkers related to centrosome replication in ulcerative colitis based on bulk transcriptome, single-cell RNA sequencing and experiments
Source: Front Immunol. 2025 Dec 12;16:1627926. doi: 10.3389/fimmu.2025.1627926 (PMC12740759; doi:10.3389/fimmu.2025.1627926)

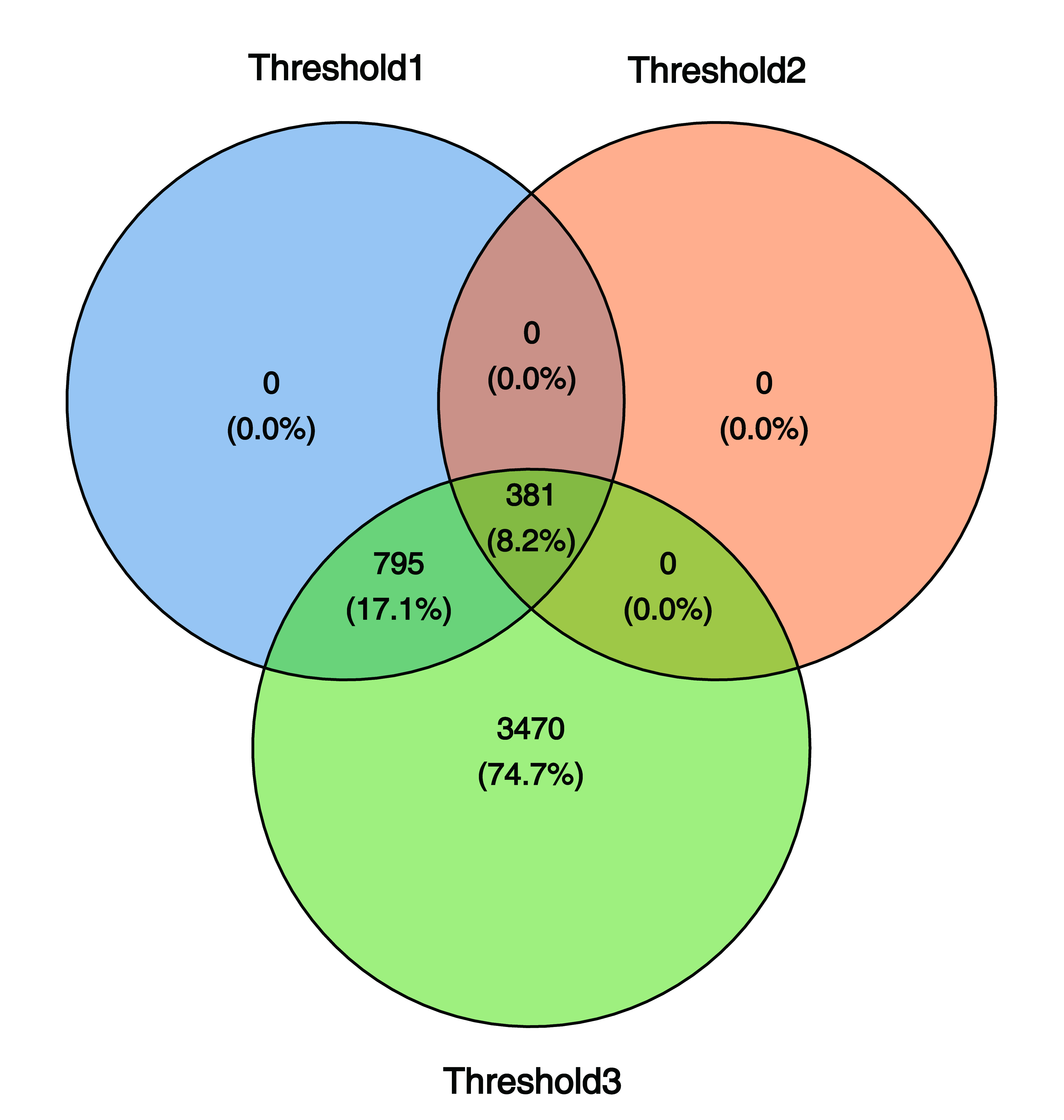

Supplement: Supplementary Figure 1 — The Venn diagram of DEGs through the intersection of three thresholds. [file Image1.tif]

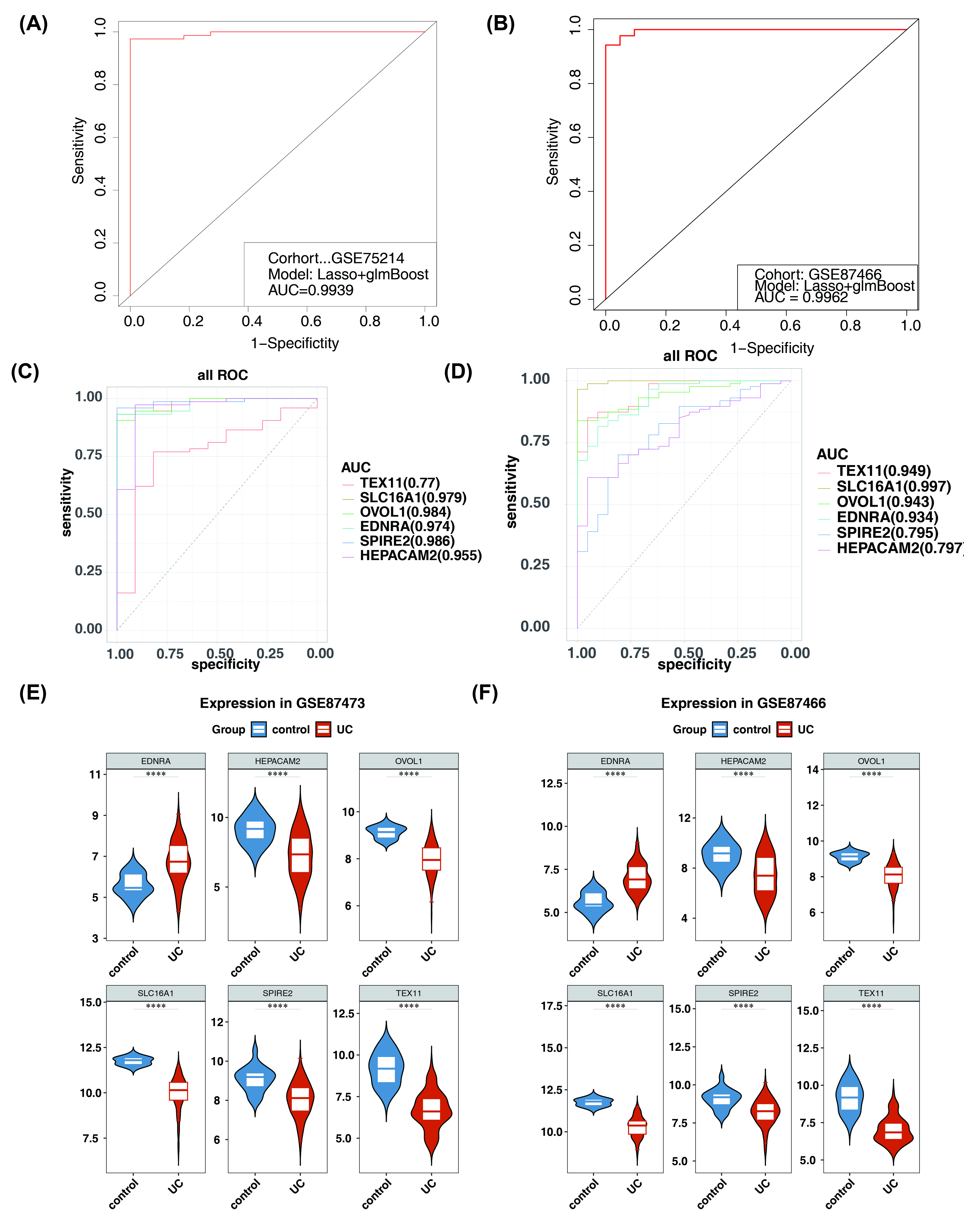

Supplement: Supplementary Figure 2 — Identification of Biomarkers. (A-B) ROC curves evaluating the accuracy of the top-performing algorithm in GSE75214 (A) and GSE87466 (B). (C-D) ROC curves for each gene in the validation sets GSE75214 (C) and GSE87466 (D). (E-F) Expression levels of candidate key genes in the validation sets GSE75214 (E) and GSE87466 (F) ROC: Receiver Operating Characteristic curve. [file Image2.tif]

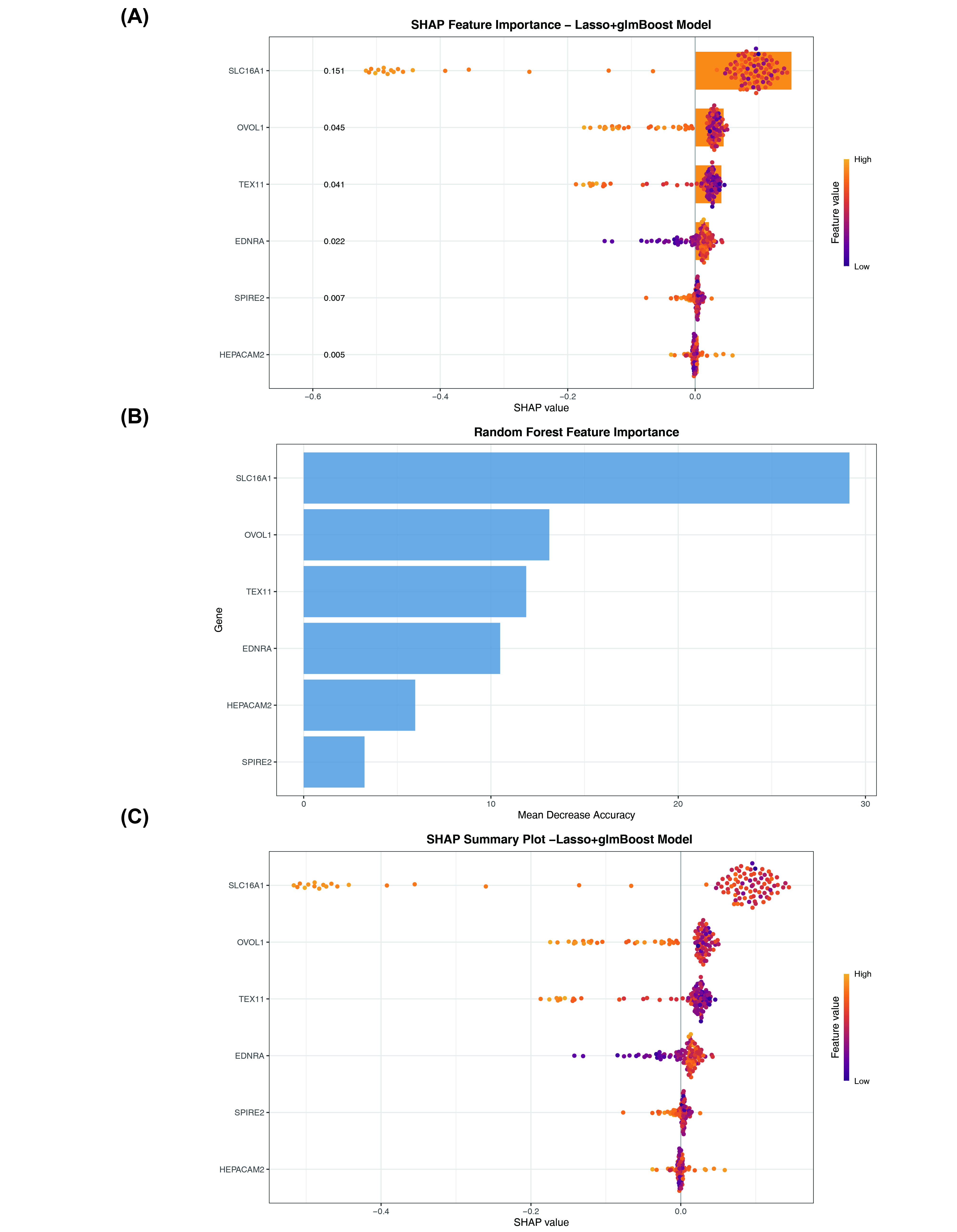

Supplement: Supplementary Figure 3 — SHAP Analysis (A) HAP feature importance plot. The x-axis represents the mean absolute SHAP value, and the y-axis lists biomarkers in descending order of importance. (B) Random forest built-in importance analysis. (C) HAP beeswarm plot. The x-axis represents the SHAP value, and the y-axis similarly shows features sorted by importance. The color of the points indicates feature expression levels (red for high expression, purple for low expression). [file Image3.tif]

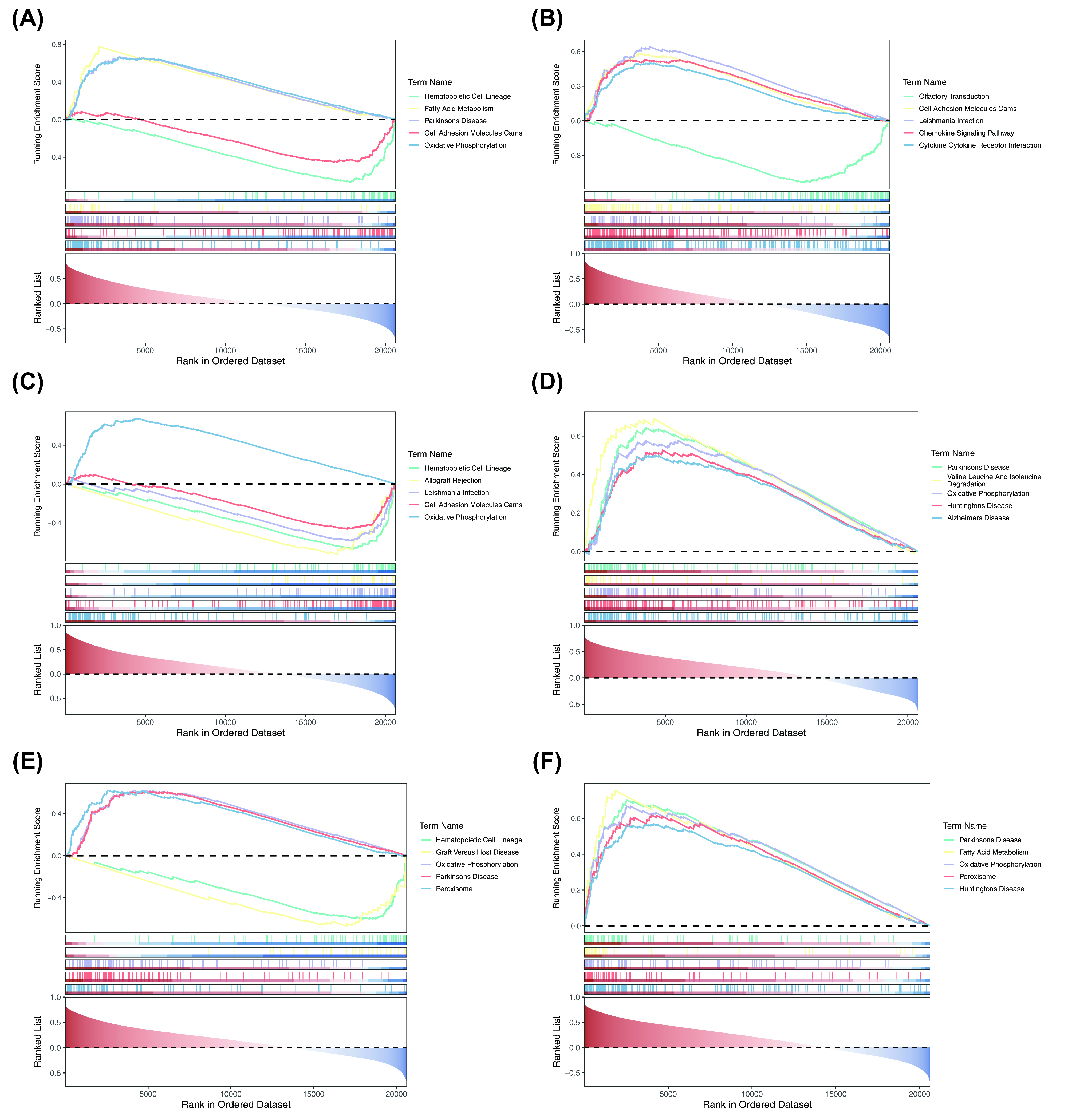

Supplement: Supplementary Figure 4 — Enrichment pathway of biomarkers. The top 5 pathways significantly enriched in biomarkers according to GSEA (A-F). The cell adhesion molecule (CAM) pathway was significantly enriched in the gene rankings associated with HEPACAM2, EDNRA, and OVOL1 (A-C), while the oxidative phosphorylation pathway was significantly enriched in the gene rankings associated with HEPACAM2, SPIRE2, TEX11, SLC16A1, and OVOL1 (A, C-F) [file Image4.tif]

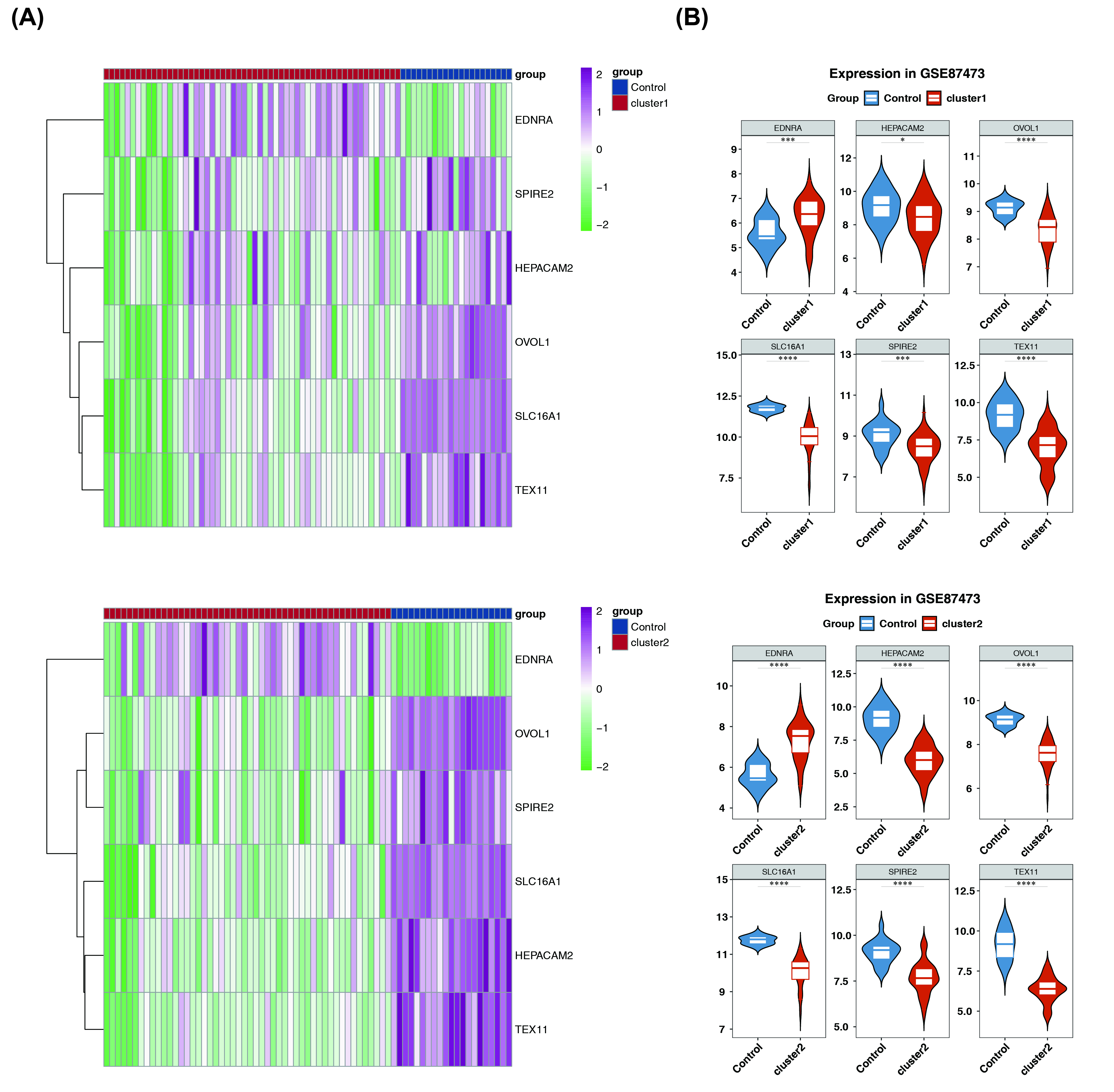

Supplement: Supplementary Figure 5 — Cluster Analysis and Expression of Key Genes in Different Subtypes. (A) Heatmap distribution of biomarkers in different clusters. (B) Boxplot of biomarker expression in different clusters. [file Image5.tif]

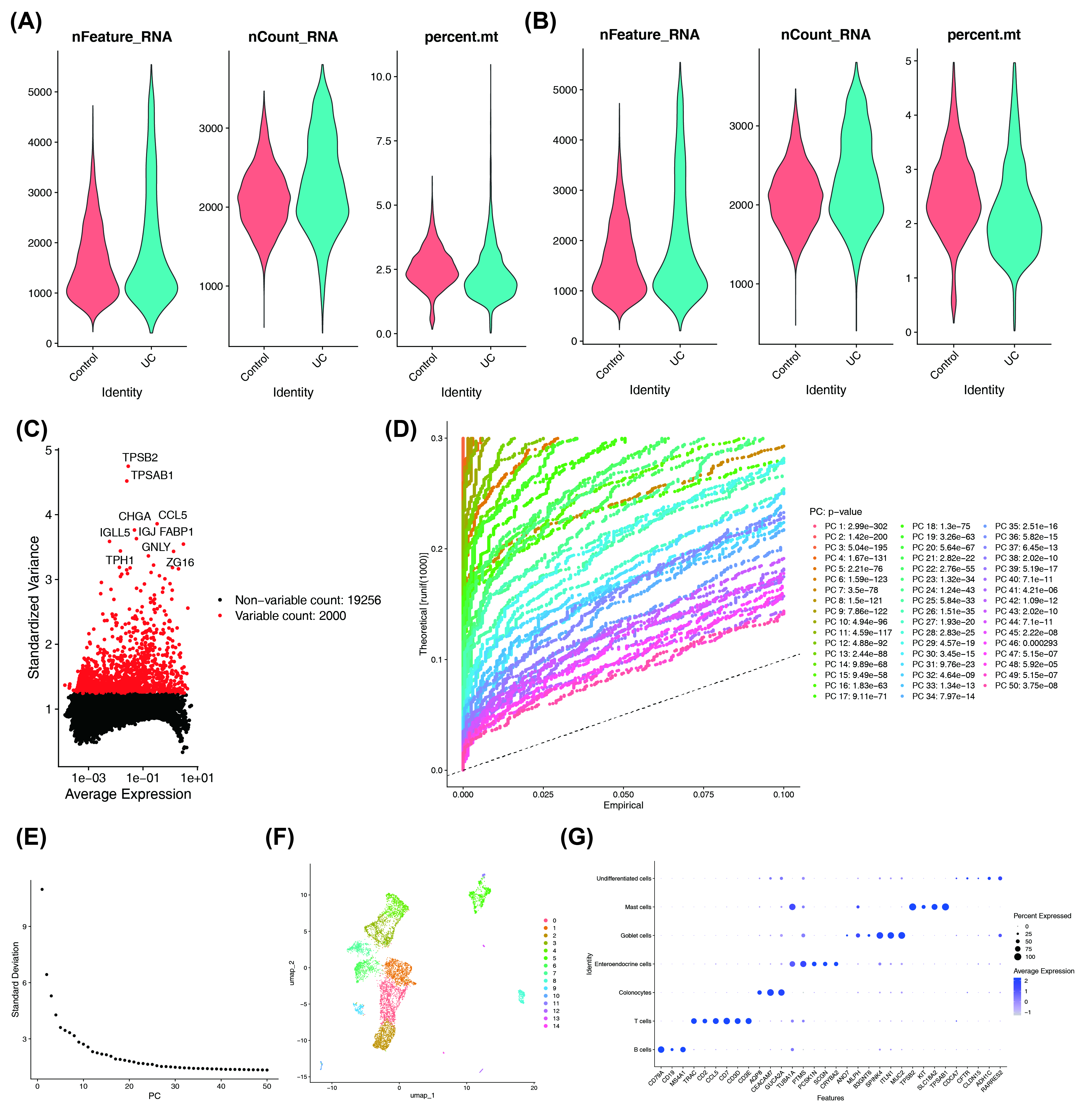

Supplement: Supplementary Figure 7 — Single-cell Analysis Data Processing and Quality Control. (A-B) Violin plots showing the distribution of nFeature_RNA, nCount_RNA, and percent.mt for sample and control samples before (A) and after (B) quality control. (C) Selection of highly variable genes. (D) JackStraw plot for Principal Component Analysis. (E) Elbow plot for Principal Component Analysis. (F) UMAP algorithm for dimensionality reduction and clustering visualization of cell clusters. (G) Bubble plot showing the expression of 7 marker genes in different cell types. [file Image7.tif]

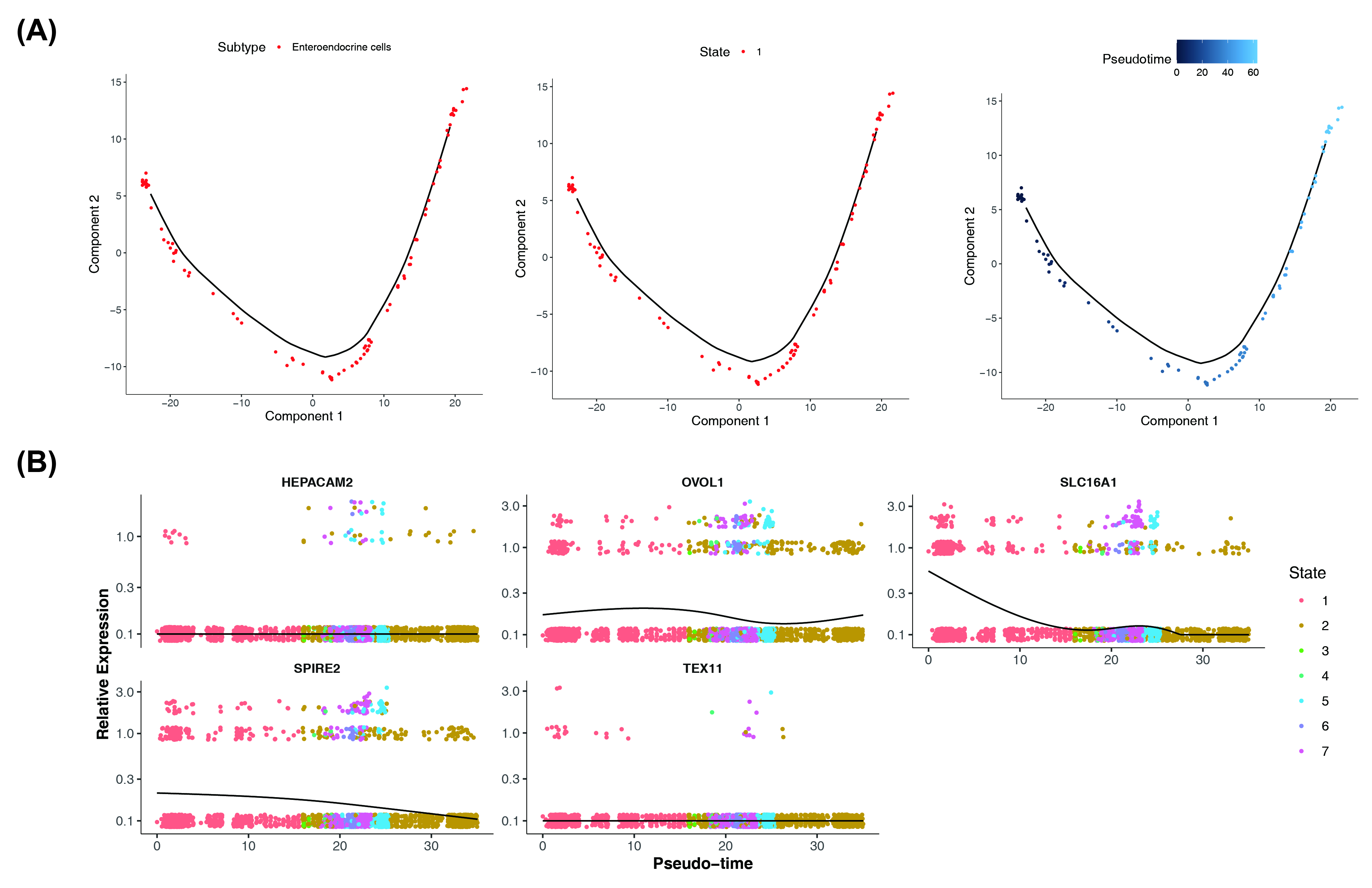

Supplement: Supplementary Figure 8 — Cell Communication Analysis and Pseudotime Analysis. (A) Differentiation trajectory of enteroendocrine cells. (B) Expression of key genes at different differentiation stages. [file Image8.tif]
